# Enzyme action optimizer based infinite impulse response filter identification through a comprehensive benchmark across full and reduced orders

**DOI:** 10.1038/s41598-025-28411-w

**Published:** 2025-11-22

**Authors:** Ridvan Firat Cinar

**Affiliations:** Faculty of Engineering and Architecture, Department of Computer Engineering, Batman University, Batman, Turkey

**Keywords:** Enzyme action optimizer, Metaheuristics, Infinite impulse response, Digital filters, Adaptive filtering, System identification, Parameter estimation, Computational biology and bioinformatics, Engineering, Mathematics and computing

## Abstract

Due to the nonlinear and multimodal nature of infinite impulse response filter coefficient spaces, achieving stable and accurate identification remains a challenging task in digital signal processing. This study aims to evaluate the capability of the recently developed bio-inspired metaheuristic algorithm, called the enzyme action optimizer, for adaptive identification of infinite impulse response (IIR) filter models with varying structural complexities. The algorithm is tested on four benchmark systems with varying levels of order and structural complexity. For each system, both full-order and reduced-order identification scenarios are examined, resulting in eight independent experiments. In every case, enzyme action optimizer is directly compared with several well-known optimization algorithms including starfish optimization algorithm, hippopotamus optimizer, and grey wolf optimization. The results are evaluated using multiple performance criteria, including mean squared error, mean absolute error, standard deviation, and convergence speed, providing a comprehensive assessment. The results clearly show that enzyme action optimizer provides highly precise identification across all cases. In low-order systems with full-order modelling, the algorithm achieves perfect reconstruction with zero mean squared error, demonstrating its ability to exactly match system dynamics. In reduced-order setups, where structural simplification introduces modelling challenges, enzyme action optimizer consistently delivers the lowest errors and most stable convergence profiles. Even in high-order and asymmetric systems, enzyme action optimizer maintains its advantage, outperforming all comparative methods both in terms of accuracy and convergence speed. The success of enzyme action optimizer stems from its biologically inspired structure, where two control parameters, adaptive factor and enzyme concentration, regulate the balance between exploration and exploitation throughout the optimization process. This dynamic control enables enzyme action optimizer to search broadly in early iterations and focus on fine-tuning in later stages. The study establishes a unified benchmarking framework that uniquely demonstrates how a biologically inspired optimizer can be effectively adapted to complex infinite impulse response identification problems, highlighting its novelty and potential for broader optimization applications.

## Introduction

### Related works on infinite impulse response filter identification and Bio-inspired optimization

In modern digital systems, where raw data is frequently noisy and intricate, digital filters stand as vital tools. They precisely shape, extract, and refine signals to isolate critical information, enhance quality, or ensure accurate system performance across applications like audio processing, communication networks, biomedical devices, and control systems. These filters generally categorized in two main classes: finite impulse response (FIR) and infinite impulse response (IIR). FIR filters ensure inherent stability and exhibit linear phase, making them ideal for applications requiring precise phase alignment. Conversely, IIR filters incorporate feedback, enabling their outputs to potentially persist indefinitely. This IIR design offers a significant advantage: it delivers sharper frequency responses with fewer components than FIR filters. This efficiency, however, introduces potential stability issues and undesired phase distortion. While FIR and IIR filters are thoroughly covered in foundational texts^1–3^, they continue to play central roles in modern signal processing applications. In particular, IIR filters are prominent in advanced digital signal processing due to their computational efficiency and their ability to model complex dynamic behaviours with fewer coefficients than FIR filters. This efficiency stems from their recursive structure, where feedback elements allow the impulse response to persist indefinitely, enabling sharper frequency responses with reduced model order. However, this advantage introduces a more intricate design process, as the feedback loop can cause nonlinearities and potential stability issues. Consequently, determining optimal IIR coefficients becomes a challenging optimization problem that requires balancing accuracy, stability, and computational cost. Because the coefficient space is nonlinear and multimodal, conventional optimization methods often struggle to reliably locate the global optimum^4^.

Recent progress points to metaheuristic algorithms as effective solutions. One such approach, using inclined planes-based methods, aims to simplify the IIR design landscape, though sometimes at the cost of global optimality^5^. Extensive comparisons of swarm-based and evolutionary algorithms, such as the Genetic Algorithm (GA), particle swarm optimization (PSO) and differential evolution (DE) show considerable improvements in error convergence and success for system identification tasks^6^.

In digital filter design workflows, the challenges related to stability and coefficient tuning lead to the adoption of chaos-inspired and opposition-based algorithms. These approaches improve the global search capabilities and help avoid local minima traps, thereby increasing the accuracy of the resulting filter coefficients^7^. Additionally, newer approaches like atom search optimization (ASO)^8,9^ and artificial hummingbird algorithm (AHA)^10^ show competitive results in estimating IIR model parameters with superior convergence behaviour and statistical performance across benchmark systems. Extensions such as parameter estimation-based IIR system identification using the improved arithmetic optimization algorithm (iaoa) have demonstrated enhanced accuracy and robustness under complex and noisy conditions^11^. A variety of nature-inspired algorithms is also explored for fractional-order IIR filters, such as the mayfly optimization algorithm, demonstrating effectiveness in fine-tuning coefficients for specific integrator orders^12^. Reduced-order modelling is similarly benefited from techniques like manta ray foraging optimization (MRFO), which balance accuracy with simplicity, especially in adaptive scenarios^13^.

From an implementation standpoint, IIR filters are particularly suited for hardware-efficient designs. Compared to FIR filters, they require fewer multipliers and enable more compact architectures, making them attractive for FPGA-based systems^14,15^. Optimization-aware digital design strategies that embed metaheuristics have proven successful in managing design complexity and reducing power consumption in such platforms^16^. In addition, recent algorithms utilizing memory-based, heap-structured, or hybrid search strategies have demonstrated high performance when applied to high-order analog and digital filter scenarios^17^. These methods offer fine control over convergence speed and solution diversity, making them fit to real-time systems where quick convergence and time efficiency is needed^18^. This growing demand for real-time applicability and resource-aware performance naturally highlights the relevance of reduced-order IIR filter structures, especially in hardware-constrained environments. Reduced-order IIR filter models are becoming popular, especially in embedded systems and portable applications where computational resources are limited^19^. These models balance performance with efficiency and show potential in applications such as hearing aid signal processing and low-power acoustic sensing^20^.

A wide variety of metaheuristic optimization algorithms are coherently applied to IIR filter design and system identification problems, owing to their strength in searching complex, nonlinear search spaces. Among the most frequently used are gravitational search algorithm (GSA)^21^, Archimedes’ optimization algorithm (AOA)^22,23^, particle swarm optimization (PSO)^24,25^ and PSO supported algorithms such as (IPSO)^26^, genetic algorithm (GA)^27,28^, collective animal behaviour based optimization algorithm (CAB)^29^, differential evolution (DE)^30,31^, and artificial bee colony (ABC)^32,33^, which are known for their simplicity and strong global search capabilities. More recent developments introduce powerful alternatives such as the improved cooperation search optimization (ICSA)^34^, teaching-learning-based optimization (TLBO)^35^, water wave algorithm^36^ and the latter often employed in hybrid frameworks to enhance exploitation. Advanced population-based algorithms like the dragonfly algorithm (DOA)^37^, sparrow search algorithm (SSA)^38^, seeker optimization algorithm (SOA)^39^, plane force system optimizer (PFSO)^40^, gravitational search algorithm (GSA)^41^ and pattern search ameliorated arithmetic optimization algorithm^42^ are also adopted due to their adaptive structure and quick convergence properties. Recent hybrid and human- or culture-inspired algorithms have also gained attention, such as the modified sine–cosine optimization for nonlinear control (MSOT)^43^, the binary anarchic society optimization (BASO)^44^, and reinforcement learning-assisted metaheuristics integrating proximal policy optimization and slime mould algorithm (PPO-SMA)^45^. These methods illustrate the expanding frontier of intelligent optimization strategies applicable to system identification and control domains.

Notably, bio-inspired strategies like the improved immune algorithm^46^, gazelle optimization algorithm (GOA)^47^ and its enhanced pair simulated annealing-based gazelle optimization algorithm (GOASA)^48^, firefly algorithm or its enhanced versions^49,50^, whale optimization algorithm (WOA)^51,52^, Chameleon swarm algorithm (CSA)^53^, pelican optimization algorithm (POA)^54^ and hybrid moth flame optimization (MFO)^55^ are shown promising results in identifying optimal IIR coefficients, particularly under noisy or high-dimensional conditions. Furthermore, the artificial rabbits optimization (ARO)^56^ and its enhanced version improved ARO (IARO)^57^ are demonstrated as competitive choices in modelling dynamic systems with reduced-order filters. These algorithms which are individually or in hybridized forms provide flexible, gradient-free optimization strategies are well-suited for real-time filtering applications where classical techniques fall short. Enhanced versions of classical optimization methods, such as genetic algorithms, have demonstrated proven performance in medical^58–60^, economics^61^ and signal processing applications^62^, providing a solid foundation and high reliability for digital filtering problems as well. Similar progress has been observed with newer hybrid metaheuristics, including the hybrid African vulture optimization algorithm (HAVOA)^63,64^, human evolutionary algorithm (HEA)^65^, and cultural history optimization algorithm (CHOA)^66^, revamped black winged kite algorithm^67^ which have demonstrated strong performance across clustering, feature selection, and engineering optimization problems.

### Enzyme action optimizer and infinite impulse response system identification

The enzyme action optimizer (EAO)^68^, recently introduced in the literature, is a bio-inspired optimization algorithm that draws inspiration from enzymatic reaction dynamics. EAO is employed in this study for its adaptive update mechanism that models enzyme–substrate interactions and dynamically controls exploration and exploitation through adjustable factors, enabling efficient optimization in complex coefficient spaces. The algorithm integrates stochastic search components with a simple regulatory mechanism and relies on a single principal parameter, which makes it computationally efficient and straightforward to implement. Previous studies have reported that EAO achieves strong performance in convergence speed and solution quality compared to other metaheuristic optimizers, suggesting its potential applicability to diverse engineering optimization problems. In recent years, IIR filter design has increasingly evolved toward nature-inspired and adaptive optimization frameworks. These methods provide advantages in terms of filter accuracy, convergence speed, and implementation cost, and have been successfully adopted in digital communication systems, audio engineering, and biomedical signal processing, fields that demand precise and computationally efficient filtering solutions.

In this study, we apply the EAO to the problem of adaptive IIR filter identification. The algorithm is systematically evaluated across four benchmark systems, each representing different filter orders and structural complexities. For each case, both full-order and reduced-order models are identified to assess the trade-off between accuracy and computational efficiency. The reduced-order scenarios are of particular interest due to their practical relevance in real-time and embedded implementations. Comparative analyses are carried out against several state-of-the-art metaheuristic algorithms reported in the literature. The experimental results indicate that the application of EAO yields competitive or superior performance in convergence behaviour and identification accuracy, confirming its suitability for practical use in signal processing and control applications.

### Contributions, novelty and future perspectives

The present study offers several contributions to the ongoing research on bio-inspired optimization by systematically examining the performance of the EAO in adaptive IIR filter identification. Building upon the established formulation of EAO, the paper aims to establish a standardized benchmark that allows fair and reproducible evaluation across multiple system complexities. Specifically, four benchmark systems with different orders and structural configurations are analysed under both full- and reduced-order settings to reveal the impact of dimensional reduction on identification accuracy and computational efficiency. The study also defines a unified experimental protocol covering population size, iteration budget, convergence criteria, and statistical reporting to ensure consistency in cross-algorithm comparison.

The main novelties and technical contributions of this study are summarized below.

- This study presents the first application of the Enzyme Action Optimizer (EAO) to the adaptive identification of Infinite Impulse Response (IIR) filters, demonstrating its capability to address nonlinear and multimodal coefficient estimation problems effectively.
- The approach is systematically evaluated under both full-order and reduced-order configurations, providing new insights into algorithmic adaptability across models with different structural complexities.
- A unified and reproducible evaluation framework is developed to compare EAO with several established metaheuristic algorithms, SFOA, HO, and GWO under identical experimental conditions. This framework introduces a comprehensive and standardized comparison strategy that has not been previously explored in the literature.
- Analytical and empirical analyses are jointly performed, encompassing convergence dynamics, identification accuracy, and computational cost to deliver a complete and transparent assessment of algorithmic performance.
- A detailed computational complexity and resource utilization study is included, revealing how EAO balances accuracy, scalability, and execution efficiency across various system dimensions.

By integrating these elements, the work provides a transparent assessment of EAO’s applicability to nonlinear, multimodal IIR identification problems and contributes a practical framework for future comparative optimization studies in signal processing.

### Organization of the study

Following the introduction, the remainder of this paper is organized as follows. "Overview of enzyme action optimizer algorithm" section provides an overview of the EAO, briefly describing its biological motivation, computational structure, and adaptive dynamics relevant to optimization. "Definition of the Problem for Infinite Impulse Response Filter Identification" formulates the IIR filter identification problem, defining the mathematical model, objective function, and associated stability constraints. "Definition of the test system setup" details the experimental setup, including the benchmark test systems, parameter configuration, and comparative algorithms used for performance evaluation. The obtained results, convergence analyses, and coefficient estimations are comprehensively presented and discussed in following subsections of the "Definition of the test system setup", followed by an analytical interpretation of the algorithm’s performance. Finally, "Discussion" concludes the study and outlines possible directions for future research.

## Overview of enzyme action optimizer algorithm

We provide a concise primer of EAO solely for completeness and refer the reader to^68^ for full algorithmic details. Any pseudocode or flow sketch used here is adapted from^68^ to clarify how EAO is plugged into our IIR identification pipeline (objective, constraints, stopping rule, parameter settings).

Evolution, swarm actions, and molecular dynamics unfold through mathematically efficient adaptations forged over millennia. These processes solve high-dimensional, uncertain, and nonlinear tasks without explicit models or gradients. Such intrinsic intelligence has inspired the design of the EAO, a metaheuristic that mimics the catalytic transformations in biochemical systems. By modelling optimization as a sequence of substrate-enzyme reactions, EAO iteratively transforms candidate solutions through biologically inspired operators. The remainder of this section details the underlying principles, computational structure, and adaptive dynamics of EAO as applied to the nonlinear identification of IIR filters.

EAO is structurally designed to replicate the sequential logic and adaptive control observed in biochemical enzyme systems, mapping these processes directly onto the domain of numerical optimization. Rather than relying solely on abstract heuristic steps, EAO formulates its search strategy around three biologically meaningful stages: initialization of candidate solutions (substrates), parameter-driven adaptive transformation (enzyme–cofactor interaction), and selective refinement toward an optimal solution (catalytic output). Each stage is grounded in both algorithmic operations and conceptual analogies from biochemistry, forming a dual-layered system where biological phenomena empower mathematical procedure.

**Fig. 1:**
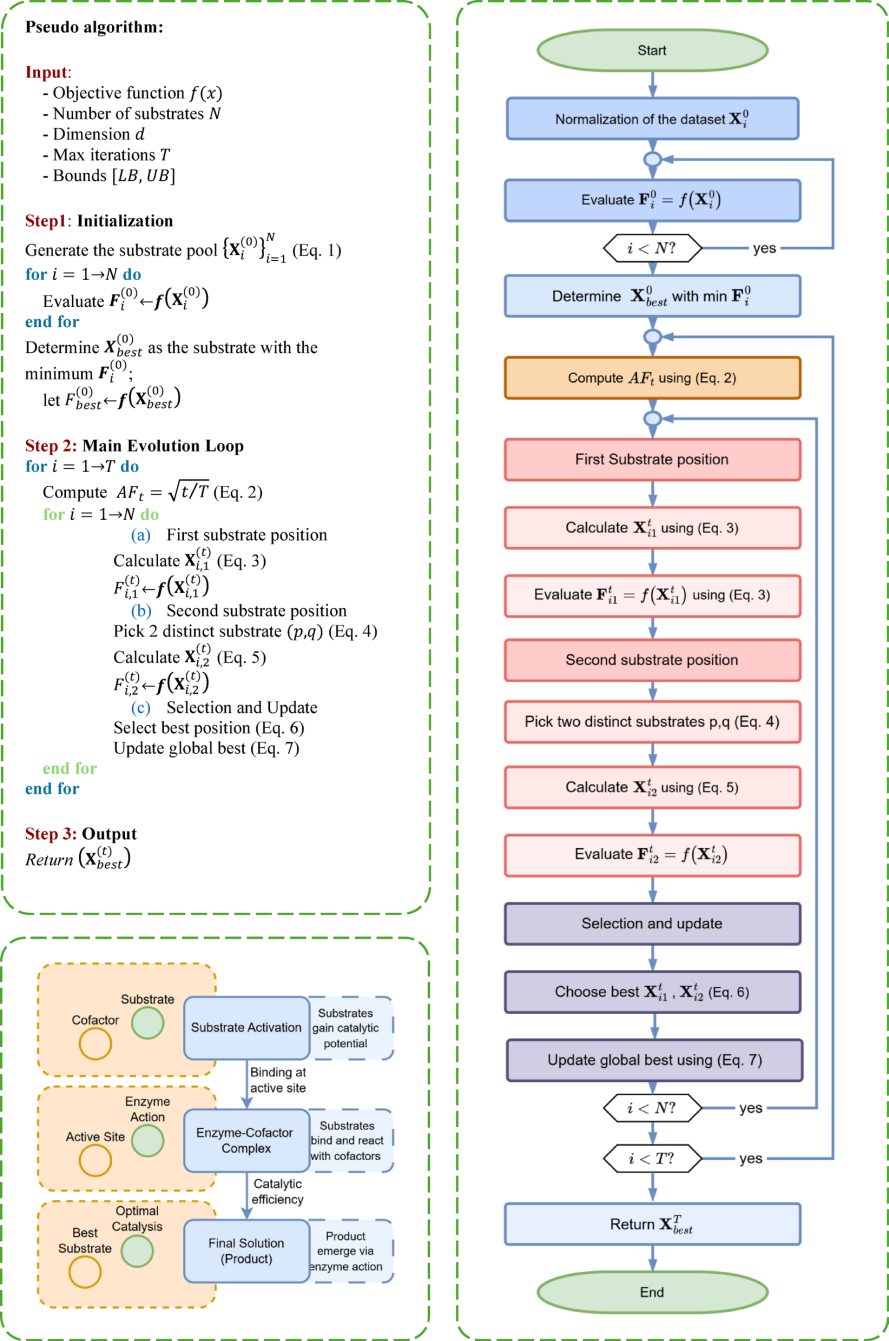
EAO overview (**a**) pseudo-code (**b**) flowchart (**c**) schematic model.

**Fig. 2:**
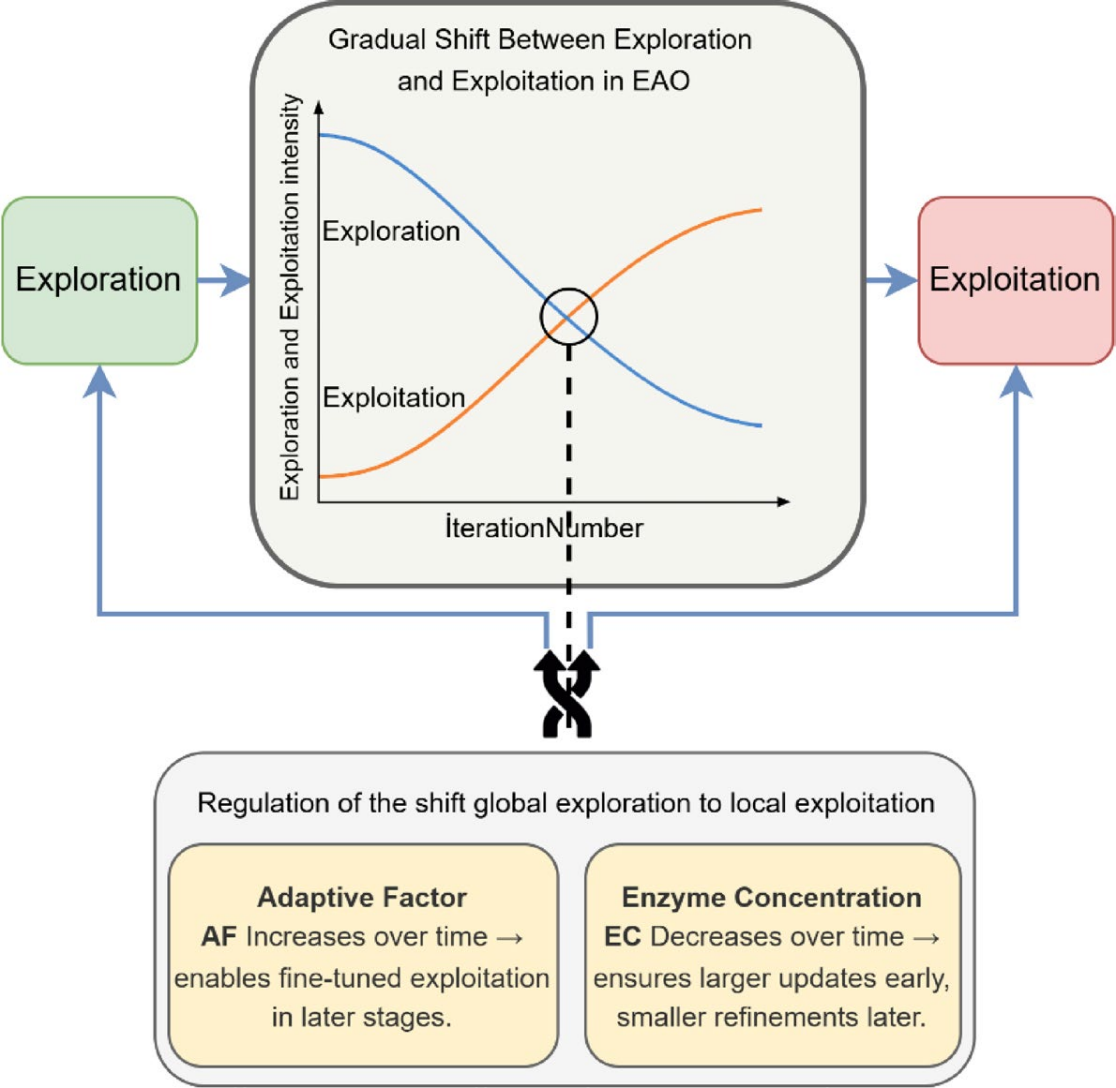
AF and EC behaviour over iterations.

This duality is clearly reflected in Fig. 1, where the pseudocode (Fig. 1.a) formalizes computational execution, the flowchart (Fig. 1.b) outlines the procedural logic, and the schematic model (Fig. 1.c) visualizes the corresponding biochemical interactions. By constructing this alignment between natural catalytic cycles and iterative optimization mechanics, EAO maintains a dynamic balance between exploration (searching diverse regions of the search space) and exploitation (refining promising areas), much like enzymes adapt their catalytic rates and substrate preferences. This balance is achieved through “cofactor-inspired control parameters” such as the adaptive factor (AF) and enzyme concentration (EC). Initially, the AF promotes exploration by enhancing population diversity, while in later stages, enzyme-substrate interactions and local refinements intensify exploitation. For a detailed schematic presentation of this case, one can examine Fig. 2.

Throughout the paper, different notations are used for clarity: scalars are represented by italic lowercase letters (e.g., *x*), vectors by bold lowercase letters (e.g., $\:\boldsymbol{x}$), and matrices by bold uppercase letters (e.g., $\:\boldsymbol{X}$). This convention is consistently applied to variables such as $\:\boldsymbol{\theta\:}$, $\:\boldsymbol{X}$, $\:y\left(t\right)$, and $\:u\left(t\right)$ for readability and distinction between mathematical entities.

The algorithm begins by randomly initializing the substrate pool using:

1 $$\:{\boldsymbol{X}}_{i}^{\left(0\right)}=LB+(UB-LB)\odot\:{r}_{i}$$

Here, $\:{\boldsymbol{X}}_{i}^{\left(0\right)}$ represents the initial position of the search agent (i.e. substrate) with the indices $\:i$, LB and UB are the lower and upper bound vectors, $\:{r}_{i}$ is a vector of regularly distributed random values in $\:[0,\:1]$, and $\:\odot\:$ denotes element-wise multiplication. The initial fitness of each substrate $\:{\boldsymbol{F}}_{i}^{\left(0\right)}$, is then evaluated using $\:{\boldsymbol{F}}_{i}^{\left(0\right)}=f\left({\boldsymbol{X}}_{i}^{\left(0\right)}\right)$, where $\:f\left(x\right)$ is the objective function to be minimized. In the main iterative loop, for each iteration $\:t$, the adaptive factor $\:\left({AF}_{t}\right)$ is calculated using

2 $$\:{AF}_{t}=\sqrt{\raisebox{1ex}{$t$}\!\left/\:\!\raisebox{-1ex}{$T$}\right.}\:\:\:,$$

where $\:T$ is the number of maximum iterations. This relationship provides a graded increase in the AF as iterations progress, shifting the algorithm from exploration to exploitation. Each substrate $\:{\boldsymbol{X}}_{i}^{\left(t-1\right)}$ generates two candidate positions. The first candidate position $\:{\boldsymbol{X}}_{i,1}^{\left(t\right)}$ is determined by:

3 $$\:{\boldsymbol{X}}_{i,1}^{\left(t\right)}=\left({\boldsymbol{X}}_{best}^{\left(t-1\right)}-{\boldsymbol{X}}_{i}^{\left(t-1\right)}\right)+{\rho\:}_{i}\cdot\:sin\left({AF}_{t}\cdot\:{\boldsymbol{X}}_{i}^{\left(t-1\right)}\right)\:\:\:$$

$\:{\boldsymbol{X}}_{best}^{\left(t-1\right)}$ is the best substrate position from the previous iteration, $\:{\boldsymbol{\rho\:}}_{\boldsymbol{i}}$ is a random vector in $\:{\left[\mathrm{0,1}\right]}^{dim}$ and the $\:sine$ function is applied elementwise. The second candidate position $\:{\boldsymbol{X}}_{i,2}^{\left(t\right)}$ is constructed by combining elements derived from two distinct substrate indices, $\:p$ and $\:q$:

4 $$\:\boldsymbol{d}={\boldsymbol{X}}_{p}^{\left(t-1\right)}-{\boldsymbol{X}}_{q}^{\left(t-1\right)}\:\:\:,$$

and is defined by:

5 $$\:{\boldsymbol{X}}_{i,2}^{\left(t\right)}={\boldsymbol{X}}_{i}^{\left(t-1\right)}+s{c}_{1}{d}_{1}+A{F}_{t}s{c}_{2}\left({\boldsymbol{X}}_{best}^{\left(t-1\right)}-{\boldsymbol{X}}_{i}^{\left(t-1\right)}\right)\:\:\:$$

The coefficients $\:s{c}_{1}$ and $\:s{c}_{2}$ which are the random scale factors within the interval $\:[EC,\:1]$, where $\:EC$ is the enzyme concentration (typically a small constant like $\:0.1$). At each iteration, both $\:{\boldsymbol{X}}_{i,1}^{\left(t\right)}$ and $\:{\boldsymbol{X}}_{i,2}^{\left(t\right)}$ are evaluated, and the better position is chosen as $\:{\boldsymbol{X}}_{l,upd}^{\left(t\right)}$. If this updated position yields improved fitness, it replaces the original substrate’s position (Eq. (8)). The global best solution, $\:{\boldsymbol{X}}_{best}^{\left(t\right)}$ is then updated if the following condition satisfied:

6 $$\:{\boldsymbol{F}(X}_{i}^{\left(t\right)})<{\boldsymbol{F}(\boldsymbol{X}}_{best}^{\left(t-1\right)})\:\:\:.$$

Furthermore, all updated substrate positions are constrained within the lower and upper bounds of the search space using:

7 $$\:{\boldsymbol{X}}_{i}\left(d\right)=max\left(min\left({\boldsymbol{X}}_{i}\left(d\right),{UB}_{d}\right),{LB}_{d}\right)\:\:\:\:,\:\:\:\forall\:d\in\:\left\{1,\:\dots\:,\:dim\right\}\:\:\:$$

This mechanism guarantees that all solutions remain within the search space boundaries throughout iterations, allowing EAO to effectively transition from broad exploration to focused refinement and converge towards optimal or near-optimal solutions.

The algorithm’s computational complexity is

8 $$\:O\left(T\cdot\:n\cdot\:\left(d+F\right)\right)\:\:\:,$$

where $\:T$ is the number of maximum iterations, $\:n$ is the number of substrates, $\:d$ is the dimensionality of each $\:{\boldsymbol{X}}_{i}$, and $\:\boldsymbol{F}$ denotes the process cost of evaluating the objective function.

## Definition of the problem for infinite impulse response filter identification

IIR systems are preferred in digital signal processing and control tasks because they capture dynamic behaviour effectively with fewer coefficients compared to FIR filters, resulting in compact and efficient models. The IIR system identification problem refers to estimating a parametric model that captures the underlying system dynamics based on observed input–output data. This task involves identifying the coefficients of a difference equation that best approximates the input–output relationship of an unknown discrete-time system.

IIR systems can typically be described by a linear constant coefficient difference equation of the following form:

9 $$\:\boldsymbol{y}\left[k\right]={\sum\:}_{j=0}^{a}{m}_{j}x\left[k-j\right]+{\sum\:}_{i=1}^{b}{n}_{i}y\left[k-i\right]\:\:\:$$

where $\:\boldsymbol{y}\left[k\right]$ is the system output at time step $\:\boldsymbol{k}$, $\:x\left[k\right]$ is the known system input, $\:{m}_{i}$ are the denominator coefficients (feedback part), $\:{n}_{j}$ are the numerator coefficients (feedforward part), $\:a$ and $\:b$ denote the orders of the denominator and numerator polynomials, respectively. In this formulation, $\:{n}_{0}=1$ is fixed to confirm uniqueness of the representation, and the recursion inherently reflects the infinite-duration impulse response of the system. System’s output at any time $\:i$ is determined by both the current and prior inputs, as well as recursively by its prior outputs.

This time-domain model corresponds to the following transfer function representation in the $\:z$-domain:

10 $$\:H\left(z\right)=\frac{B\left(z\right)}{A\left(z\right)}=\frac{{m}_{0}+{m}_{1}{z}^{-1}+\dots\:+{m}_{b}{z}^{-b}}{1-{n}_{1}{z}^{-1}-\dots\:-{n}_{a}{z}^{-a}}\:\:\:$$

where $\:B\left(z\right)$ is the numerator polynomial capturing the input effect and $\:A\left(z\right)$ is the denominator polynomial representing the system memory. The objective in IIR system identification is to estimate the vectors $\:{\boldsymbol{\theta\:}}_{n}=\left[{n}_{1},{n}_{2},\dots\:,{n}_{a}\right]$ and.

**Fig. 3:**
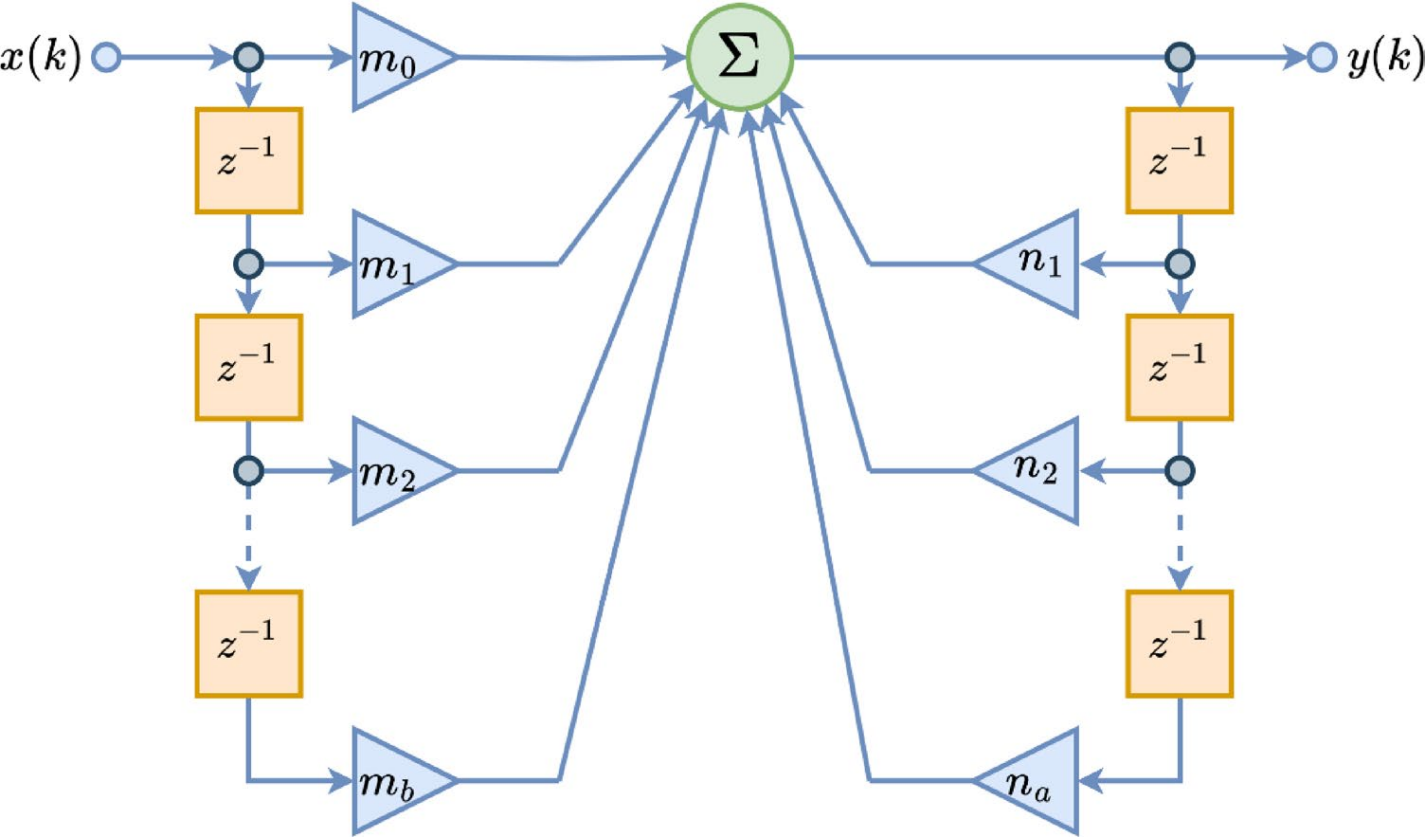
Schematic representation of IIR filter.

$\:{\boldsymbol{\theta\:}}_{m}=\left[{{m}_{0},m}_{1},{m}_{2},\dots\:,{m}_{b}\right]$ such that the model output $\:y\left[k\right]$ is as close as possible to the observed output $\:d\left[k\right]$, which is assumed to come from the real system. Figure 3 can be examined for a better understanding on IIR filter structure that forward and backward operations seen clearly.

In some applications, especially in embedded or constrained environments, reduced-order models are preferred for real-time implementation. This approach aims to approximate the true system using a model of lower order,

11 $$\:\widehat{a}<\stackrel{-}{a}\:\:\:and\:\:\:\:\widehat{b}<\stackrel{-}{b}\:\:\:$$

where the estimated numerator order ($\:\widehat{a}$) is less than the true numerator order ($\:\stackrel{-}{a}$), and similarly, the estimated denominator order ($\:\widehat{b}$) is less than the true denominator order ($\:\stackrel{-}{b}$). This simplification introduces an additional trade-off between the model’s simplicity and its accuracy in representing the original system.

## Definition of the test system setup

In this section, the theoretical framework described in "Definition of the Problem for Infinite Impulse Response Filter Identification" is applied to the IIR identification problem. The optimization variable $\:\boldsymbol{\theta\:}$ represents the IIR coefficients defined in (10), and the cost function $\:MSE\left(\boldsymbol{\theta\:}\right)$, given in (12), serves as the performance metric minimized by EAO during each iteration. The algorithm evaluates $\:MSE\left(\boldsymbol{\theta\:}\right)$ through forward simulation of the model output $\:y(k,\boldsymbol{\theta\:})$, followed by stability enforcement and coefficient updates as described previously.

**Fig. 4:**
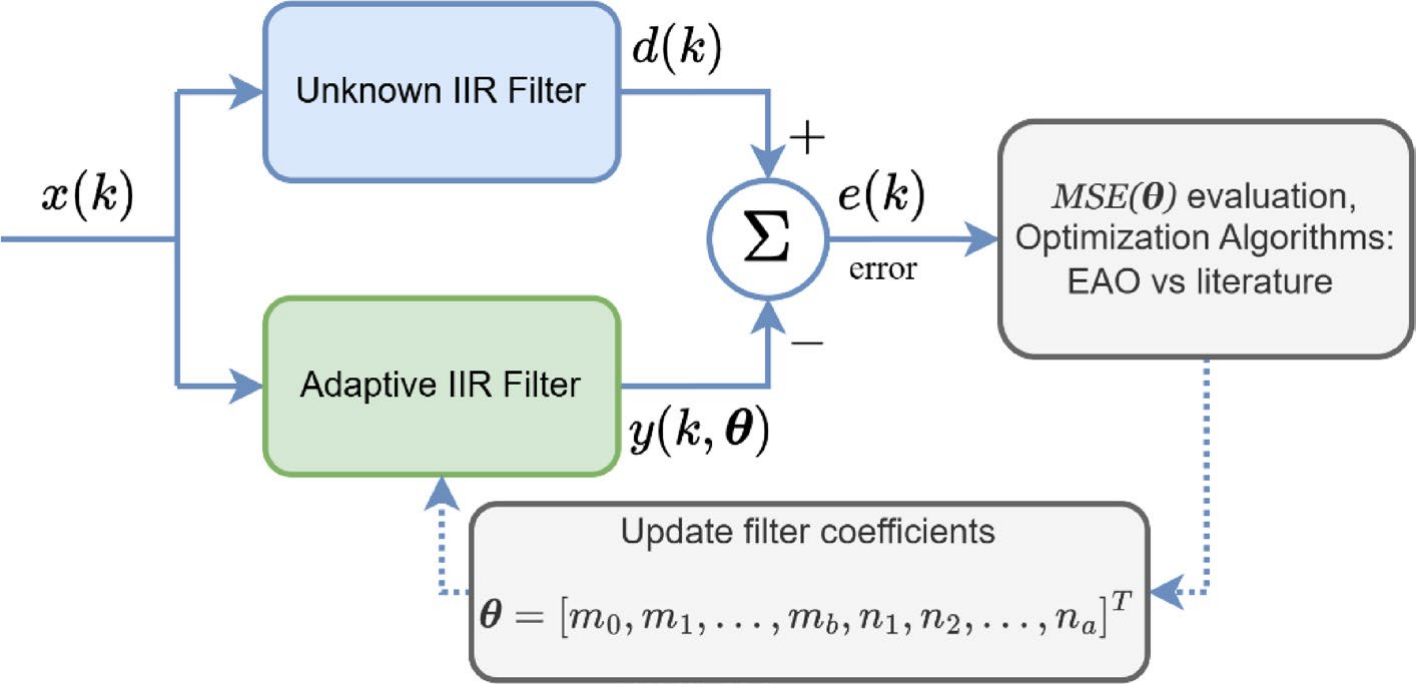
Schematic flow of the IIR test system.

As illustrated in Fig. 4, the IIR filter identification problem is modelled as an iterative optimization task, where the objective is to adjust the coefficient seen in numerator and denominator of an adaptive IIR filter’s mathematical expression so that its output $\:y\left(k\right)$ closely matches that of an unknown reference filter $\:d\left(k\right)$, both driven by a shared input signal. The difference between these two outputs at each discrete time step $\:k$ yields the error signal $\:e\left(k\right)$, which quantifies the instantaneous modelling discrepancy. The optimization algorithm evaluates this error across a finite observation window and seeks the parameter vector $\:\boldsymbol{\theta\:}$ that minimizes the mean squared error, typically expressed as in (12).

In summary, the optimization problem is formulated as minimizing the mean squared error (MSE) between the reference output and the adaptive IIR model output with respect to the coefficient vector, subject to stability constraints that ensure all poles remain within the unit circle.

12 $$\:MSE\left(\boldsymbol{\theta\:}\right)=\frac{1}{N}{\sum\:}_{k=1}^{N}{e}^{2}\left(k\right)=\frac{1}{N}{\sum\:}_{k=1}^{N}{\left[d\left(k\right)-y\left(k,\boldsymbol{\theta\:}\right)\right]}^{2}\:\:\:$$

Here, $\:y(k,\boldsymbol{\theta\:})$ denotes the adaptive filter output computed using the current parameter set $\:\boldsymbol{\theta\:}$, in another words the vector consisting of the coefficients in IIR studies, and $\:d\left(k\right)$ is the reference output produced by the unknown IIR filter. The optimization engine, such as EAO, GWO, SFAO, HO, iteratively updates $\:\boldsymbol{\theta\:}$ vector to reduce this error, guiding the adaptive filter to replicate the time-domain behaviour of the reference system. Since the adaptive filter includes recursive feedback, the dependency of $\:d\left(k\right)$ on past outputs introduces nonlinearity into the error surface, making global optimization methods especially suitable. Over successive iterations, the adaptive model aligns its dynamics with the unknown system, ultimately minimizing $\:e\left(k\right)$ and achieving accurate parameter identification.

Moreover, for practical use, the filter must be stable, meaning all the poles of $\:H\left(z\right)$ (i.e., roots of $\:A\left(z\right)$) must lie strictly inside the unit circle in the complex plane. This introduces additional constraints during the identification process. Given the complexity of the cost surface and stability constraints, heuristic and data-driven approaches are often adopted in practical implementations.

Table 1 outlines the test systems employed to mathematically evaluate the effectiveness of the presented method for identifying IIR filters. Four different systems are stated, each characterized by a base transfer function that serves as the reference model. For every system, two modelling configurations are considered: full-order and reduced-order models. In the full-order case, the structure of the adaptive IIR model matches that of the reference system, allowing for exact identification in ideal conditions. The reduced-order case, on the other hand, imposes a structural simplification by lowering the number of coefficients, introducing a trade-off between model complexity and approximation accuracy.

**Table 1:** Test systems and coefficients to be optimized.

Test identity		Transfer function
Test system number I	Reference model	$\:{H}_{b}\left(z\right)=\frac{0.05-0.4{z}^{-}1}{1-1.1314{z}^{-1}+{0.25z}^{-2}}$
	Same order	$\:{H}_{s}\left(z\right)=\frac{{m}_{0}+{m}_{1}{z}^{-1}}{1-{n}_{1}{z}^{-1}-{n}_{2}{z}^{-2}}$
	Reduced order	$\:{H}_{r}\left(z\right)=\frac{{m}_{0}}{1-{n}_{1}{z}^{-1}}$
Test system number II	Reference model	$\:{H}_{b}\left(z\right)=\frac{-0.2-0.4{z}^{-1}+{0.5z}^{-2}}{1-0.6{z}^{-1}+{0.25z}^{-2}-{0.2z}^{-3}}$
	Same order	$\:{H}_{s}\left(z\right)=\frac{{m}_{0}+{m}_{1}{z}^{-1}+{m}_{2}{z}^{-2}}{1-{n}_{1}{z}^{-1}-{n}_{2}{z}^{-2}-{n}_{3}{z}^{-3}}$
	Reduced order	$\:{H}_{r}\left(z\right)=\frac{{m}_{0}+{m}_{1}{z}^{-1}}{1-{n}_{1}{z}^{-1}-{n}_{2}{z}^{-2}}$
Test system number III	Reference model	$\:{H}_{b}\left(z\right)=\frac{1-0.9{z}^{-1}+{0.81z}^{-2}-{0.729z}^{-3}}{1+0.04{z}^{-1}+{0.2775z}^{-2}-{0.2101z}^{-3}+0.14{z}^{-4}}$
	Same order	$\:{H}_{s}\left(z\right)=\frac{{m}_{0}+{m}_{1}{z}^{-1}+{m}_{2}{z}^{-2}+{m}_{3}{z}^{-3}}{1-{n}_{1}{z}^{-1}-{n}_{2}{z}^{-2}-{n}_{3}{z}^{-3}-{n}_{4}{z}^{-4}}$
	Reduced order	$\:{H}_{r}\left(z\right)=\frac{{m}_{0}+{m}_{1}{z}^{-1}+{m}_{2}{z}^{-2}}{1-{n}_{1}{z}^{-1}-{n}_{2}{z}^{-2}-{n}_{3}{z}^{-3}}$
Test system number VI	Reference model	$\:{H}_{b\left(z\right)}=\frac{0.1084+0.5419{z}^{-1}+1.0837{z}^{-2}+1.0837{z}^{-3}+0.5419{z}^{-4}+0.1084{z}^{-5}}{1+0.9853{z}^{-1}+0.9738{z}^{-2}+0.3864{z}^{-3}+0.1112{z}^{-4}+-0.0113{z}^{-5}}$
	Same order	$\:{H}_{s}\left(z\right)=\frac{{m}_{0}+{m}_{1}{z}^{-1}+{m}_{2}{z}^{-2}+{m}_{3}{z}^{-3}+{m}_{4}{z}^{-4}+{m}_{5}{z}^{-5}}{1-{n}_{1}{z}^{-1}-{n}_{2}{z}^{-2}-{n}_{3}{z}^{-3}-{n}_{4}{z}^{-4}-{n}_{5}{z}^{-5}}$
	Reduced order	$\:{H}_{r}\left(z\right)=\frac{{m}_{0}+{m}_{1}{z}^{-1}+{m}_{2}{z}^{-2}+{m}_{3}{z}^{-3}+{m}_{4}{z}^{-4}}{1-{n}_{1}{z}^{-1}-{n}_{2}{z}^{-2}-{n}_{3}{z}^{-3}-{n}_{4}{z}^{-4}}$

**Fig. 5:**
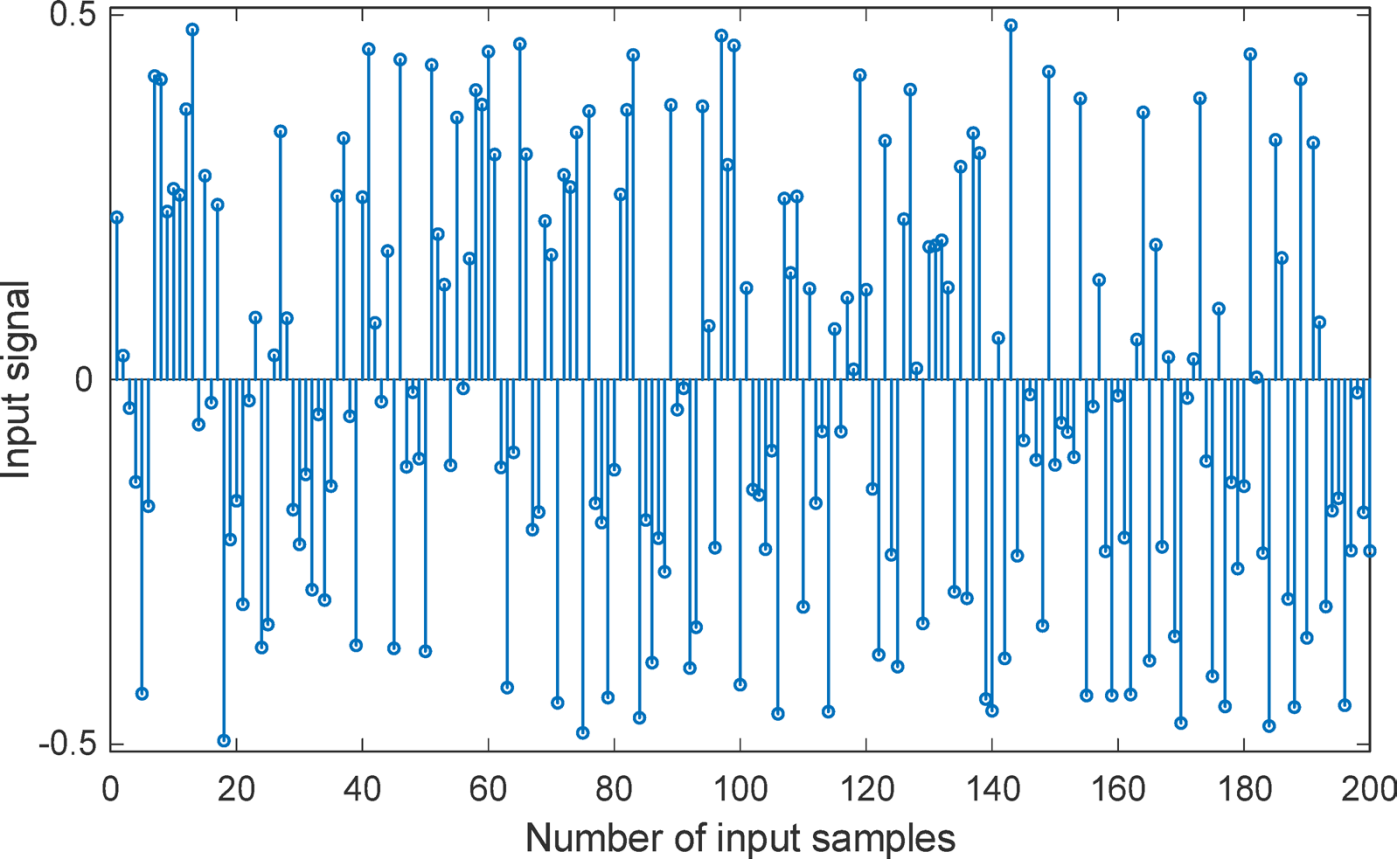
Input signal that commonly used in various tests.

Figure 5 illustrates the 200-sample long input signal used throughout the identification experiments for all test systems, which are considered as widely used benchmark examples in the literature. The signal is designed to sufficiently excite the dynamic behaviour of both the reference and adaptive IIR filters across a wide frequency range. This ensures that the system’s full response characteristics, both transient and steady state are observable and learnable during optimization. By applying the same input to each model, consistency is maintained across tests, allowing for fair comparison of algorithm performance. The choice of input structure is critical, as insufficient excitation can lead to incomplete parameter estimation or convergence to local minima.

To ensure fairness and comparability across all optimization algorithms, a standardized experimental setup is adopted. Each algorithm is executed for 30 independent runs, each consisting of 1000 iterations, with a population size fixed at 50 individuals. A preliminary sensitivity check was performed for EAO’s control parameters (AF and EC) to confirm that their adaptive ranges yield stable performance across all benchmark systems. These settings, together with uniform population size, iteration limit, and stopping criteria across all algorithms, ensure a fair and reproducible evaluation framework. This configuration provides a consistent statistical basis (mean, standard deviation, best, and worst values) and enables meaningful comparisons among all methods. Here, the control parameters are the AF and the EC. These are user-defined parameters that regulate the balance between exploration and exploitation throughout the optimization process. In all experiments, AF was varied within $\:[0.2,\:0.9]$ and EC within $\:[0.1,\:0.8]$, both linearly adapted across iterations to promote global exploration at the beginning and fine-tuning toward convergence in later stages. Randomness in EAO arises only from initial population generation and stochastic update operations performed in each iteration. All other settings, including population size, iteration budget, and stopping criteria, are kept identical across algorithms to ensure a fair and reproducible evaluation framework.

In each test system, the EAO is evaluated alongside several well-established metaheuristic algorithms that have previously demonstrated strong performance in similar identification tasks. The goal is to conduct a comparative analysis under identical experimental conditions, focusing on criteria such as identification accuracy, convergence speed, and model fidelity. By benchmarking EAO against these reference algorithms, the study aims to assess its effectiveness across varying system dynamics and structural configurations. The following sections present the results of these comparisons and provide a basis for interpreting the relative performance of each method.

### Test system I: second order infinite impulse response filter and reduced order equivalent

Test System I is simply defined by a second-order IIR filter with both feedforward and feedback components. The system consists of two numerator coefficients and two denominator coefficients, forming a standard bi-quad structure as in (13). In the full-order model in (14), the filter uses the same number of coefficients as the reference system, allowing for exact structural representation. In the reduced-order case formulized in (15), one feedback and one feedforward term are removed in order to simplify the model. This system represents a relatively low-complexity scenario, making it suitable for evaluating the baseline identification performance of the algorithms. This test is set to evaluate how accurately the adaptive model captures the system dynamics represented by the coefficients given below, under both filter structures.

13 $$\:{H}_{1}\left({z}^{-1}\right)=\frac{{m}_{0}+{m}_{1}{z}^{-1}}{1-{n}_{1}{z}^{-1}-{n}_{2}{z}^{-2}}\:\:\:$$

where coefficients considered for the test system are $\:{m}_{0}=0.05$, $\:{m}_{1}=-0.4$, $\:{n}_{1}=1.1314$ and $\:{n}_{2}=-0.25$

14 $$\:{H}_{1,\:full}\left({z}^{-1}\right)=\frac{{\delta\:}_{0}+{\delta\:}_{1}{z}^{-1}}{1-{\theta\:}_{1}{z}^{-1}-{\theta\:}_{2}{z}^{-2}}\:\:\:$$

15 $$\:{H}_{1,\:reduced}\left({z}^{-1}\right)=\frac{{\delta\:}_{0}}{1-{\theta\:}_{1}{z}^{-1}}\:\:\:$$

To evaluate the performance of the presented method, the EAO^68^ is compared against three other optimization algorithms: the starfish optimization algorithm (SFOA)^69^ and the hippopotamus optimizer (HO)^70^, which represent recent developments in metaheuristic design, and the grey wolf optimizer (GWO)^71^, a well-established algorithm frequently used in system identification studies. These algorithms are selected to provide a balanced comparison between contemporary and widely adopted techniques.

#### Full-order system

**Table 2:** Numerical results of MSE values for test system I in case of full-order system.

Algorithm	Minimum	Maximum	Average	Standard deviation	Rank
EAO	0	0	0	0	1
SFOA	2.8598E − 20	1.1836E − 17	1.2851E − 18	2.4924E − 18	2
HO	3.6336E − 08	2.4379E − 04	4.5981E − 05	6.3293E − 05	3
GWO	6.0315E − 09	1.6746E − 02	6.9291E − 04	3.0476E − 03	4

Table 2 clearly shows the outstanding performance of the EAO on the full-order configuration of Test System I. Remarkably, EAO achieved a zero-mean squared error ($\:MSE=0$) across all 30 independent runs. Here, technical meaning of the zero is the algorithm is able to perfectly match the true system model with no discrepancy at all between the reference and identified outputs. In practical terms, this indicates that EAO reaches the global optimum consistently and did so with exceptional stability and precision. While the other algorithms (SFOA, HO, and GWO) provide reasonable results, they still show some variation and error, especially when compared to EAO’s flawless performance. These findings suggest that EAO is not only a competitive alternative but potentially a benchmark optimizer for this type of IIR system identification problem.

**Table 3:** Optimal coefficients of test system I for full-order system configuration.

Algorithm	$\:{\theta\:}_{1}$	$\:{\theta\:}_{2}$	$\:{\delta\:}_{0}$	$\:{\delta\:}_{1}$
EAO	1.1314	−0.2500	0.0500	−0.4000
SFOA	1.1314	−0.2500	0.0500	−0.4000
HO	1.1305	−0.2492	0.0494	−0.3997
GWO	1.1310	−0.2497	0.0501	−0.4003

Table 3 shows the optimal coefficients obtained by each algorithm for the full-order model of Test System I. The results show that EAO and SFOA are both able to exactly recover the true parameter values of the reference system, achieving a perfect match for all four coefficients ($\:{\theta\:}_{1}\:,{\theta\:}_{2}\:,{\delta\:}_{0}\:,{\delta\:}_{1}$). This confirms that both algorithms are capable of accurately navigating the error surface and locating the precise global solution under ideal conditions. However, while SFOA matched the values in this specific case, it is not able to achieve zero error in Table 2, suggesting that its convergence may be more sensitive to initials or tend to occasional local trapping. In contrast, EAO’s steady accuracy across all runs highlights not only its ability to find the correct parameters, but also its reliability in doing so. HO and GWO, although close, introduced small deviations in the coefficient values, reflecting minor estimation errors and a less precise modelling outcome.

**Fig. 6:**
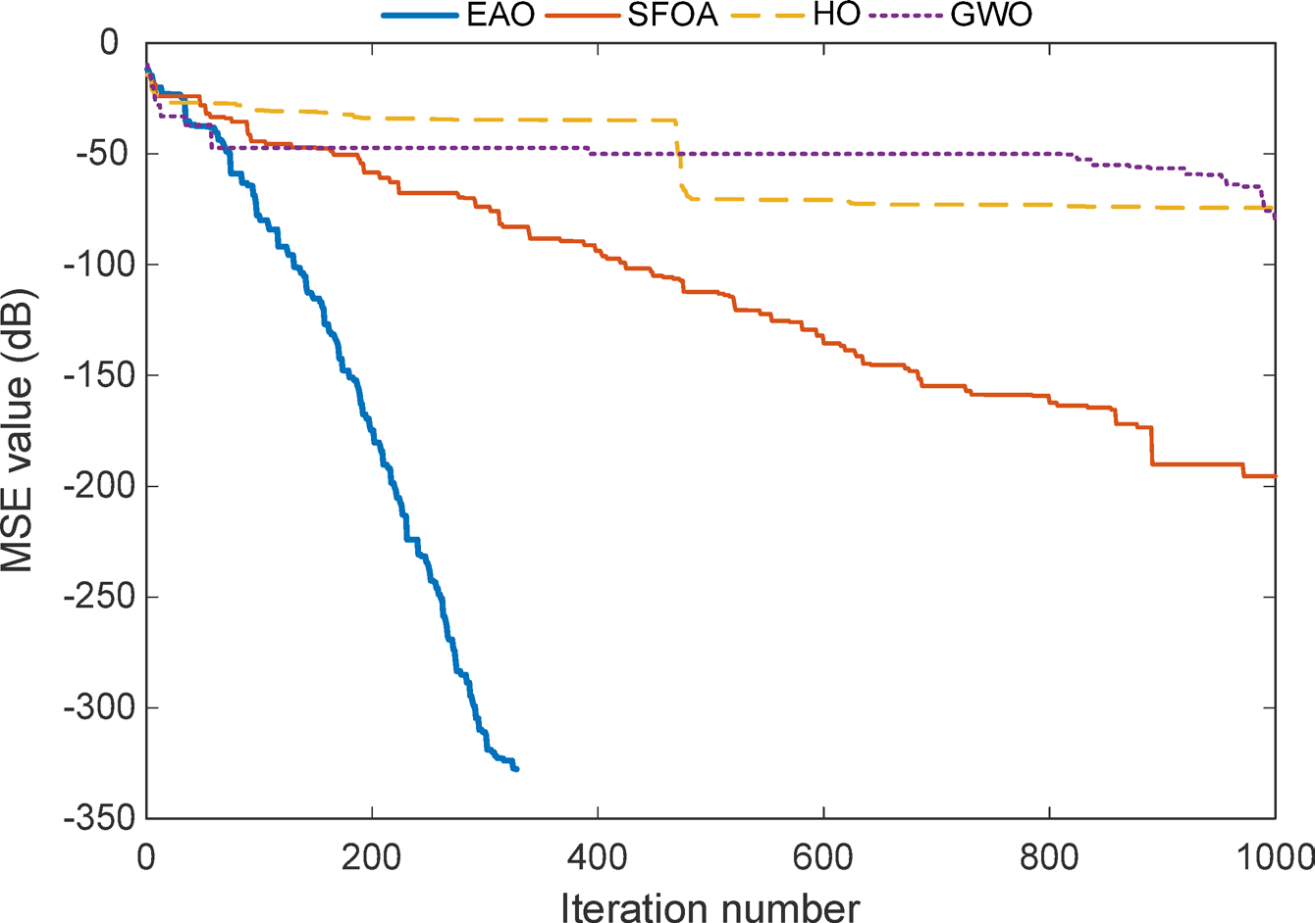
Convergence curve of the full-order model for Test System I.

The convergence curve for Test System I seen in Fig. 6, shows a clear and detailed summary of how each algorithm progresses toward the optimal solution over the sequence of iterations. When we observe the plot, one of the most obvious features is the performance of the EAO. Among given algorithms, EAO reaches a near-zero error level in approximately 330 iterations, as reflected by the sharp drop in its curve. This rapid and smooth descent indicates both fast convergence and high stability, which strongly supports the results previously shown in Tables 2 and 3 that EAO achieved zero MSE and exact coefficient recovery.

The graph uses a logarithmic scale in dB to display MSE values, which helps to emphasize small error differences. In this context, EAO’s curve plunges down to around − 300 dB, which corresponds to an extremely small numerical error (on the order of $\:{10}^{-30}$). This shows not only a successful convergence but also a mathematically precise solution, approaching the numerical limits of the computing environment.

In contrast, the other algorithms, SFOA, HO, and GWO exhibit slower or less reliable convergence behaviour. SFOA improves gradually but plateaus early, while HO and GWO show even more limited progress. This difference reflects what is also seen in the statistical results: although SFOA sometimes finds correct coefficients, its error does not always drop to zero, possibly due to instability or sensitivity to initial conditions. HO and GWO show small coefficient deviations and larger MSE values overall, confirming their less accurate modelling performance.

#### Reduced-order system

In the reduced-order model, one feedforward and one feedback term are intentionally removed, which simplifies the structure and lowers the model’s order. This simplification is often preferred in real-time or resource-constrained applications, as it reduces computational cost and memory usage. Yet, it comes with a trade-off: while the model becomes more efficient, it may lose some accuracy in capturing the system’s full dynamic behaviour. The comparison between both configurations allows us to evaluate not only how well each algorithm performs under ideal conditions, but also how strong they are when the system complexity is reduced.

**Table 4:** Numerical results of MSE values for test system I in case of reduced-order system.

Algorithm	Minimum	Maximum	Average	Standard deviation	Rank
EAO	1.1122E − 02	1.2614E − 02	1.1954E − 02	3.7767E − 04	1
SFOA	1.1939E − 02	1.3271E − 02	1.2594E − 02	3.2133E − 04	3
HO	1.1531E − 02	1.2904E − 02	1.2314E − 02	3.6122E − 04	2
GWO	1.1808E − 02	2.2356E − 02	1.3811E − 02	2.7285E − 03	4

**Table 5:** Optimal coefficients of test system I for reduced-order system configuration.

Algorithm	$\:{\theta\:}_{1}$	$\:{\delta\:}_{0}$
EAO	0.9141	– 0.2677
SFOA	0.9244	– 0.3287
HO	0.8977	– 0.3144
GWO	0.9018	– 0.3029

Tables 4 and 5 present the performance of each algorithm in estimating the parameters of the reduced-order model for Test System I. Although this setup simplifies the original system by removing one numerator and one denominator term, it still poses a challenge for accurate system representation. Despite this structural limitation, the EAO delivers the best overall performance, achieving the lowest MSE among all compared algorithms. As shown in Table 4, EAO maintains a small error range and the lowest standard deviation, indicating both precision and consistency across runs. In Table 5, the estimated coefficients obtained by EAO are also the closest to the reference system, outperforming SFOA, HO, and GWO. This highlights EAO’s ability to adapt effectively to simplified models and maintain high identification quality even when perfect reconstruction is not structurally possible. These results demonstrate that EAO is not only powerful in full-order scenarios but also strong and reliable under reduced-order conditions, where many algorithms tend to struggle.

**Fig. 7:**
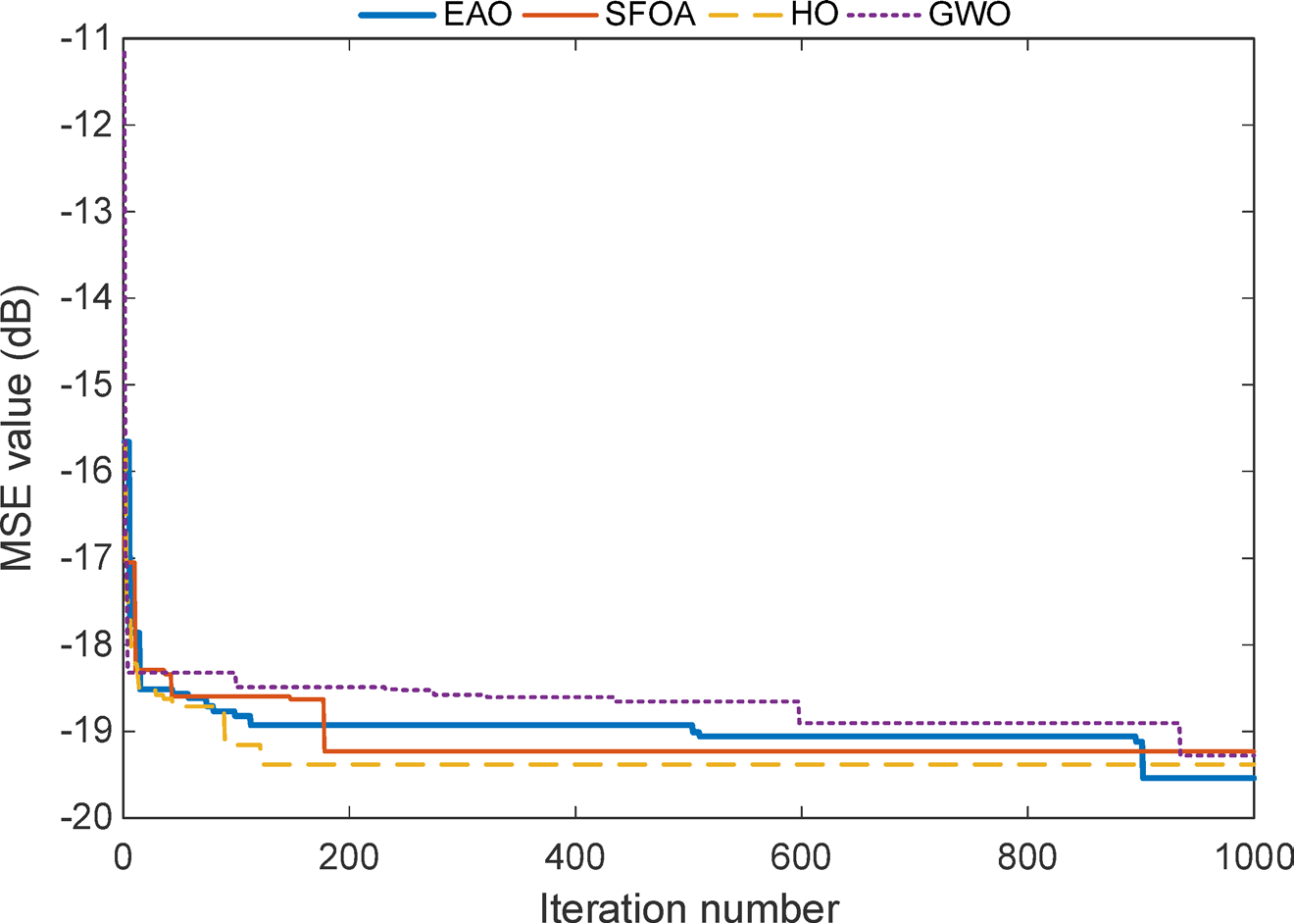
Convergence curve of the reduced-order model for Test System I.

The convergence curve seen in Fig. 7 for the reduced-order model of Test System I reveals a much slower and flatter progression compared to the full-order case. While all algorithms show initial improvements within the first 200 iterations, the most significant changes occur around iteration 850, where EAO makes a final refinement that pushes the MSE value below − 19.5 dB. This late improvement indicates that, even in a simplified model structure, EAO continues to search and fine-tune the solution space, ultimately achieving the most optimal result. In contrast, SFOA, HO, and GWO converge earlier but plateau at slightly higher error levels, suggesting that they stabilize prematurely or settle near local minima. Overall, this curve confirms EAO’s long-term optimization strength, proving its ability to maintain active search behaviour and outperform competitors even when early convergence seems similar.

#### Comparison with reported works

**Table 6:** Performance comparison of EAO with State-of-the-Art algorithms in Full-Order and Reduced-Order modelling of test system I.

Model type	Algorithm	Minimum	Maximum	Average	Standard deviation	Rank
Full-order	EAO	0	0	0	0	1
	ASO	6.1032E − 26	3.4896E − 23	8.5799E − 24	8.9133E − 24	2
	MFO	4.6466E − 15	6.0558E − 08	7.7854E − 09	1.5518E − 08	3
	GSA	3.1619E − 05	2.0703E − 03	5.2445E − 04	4.8648E − 04	5
	ABC	3.3849E − 06	1.3398E − 04	2.2365E − 05	2.4763E − 05	4
Reduced-order	EAO	1.1122E − 02	1.2614E − 02	1.1954E − 02	3.7767E − 04	1
	ASO	1.1436E − 02	1.2390E − 02	1.2115E − 02	2.3867E − 04	2
	MFO	1.1566E − 02	1.2714E − 02	1.2249E − 02	2.7452E − 04	3
	GSA	1.2054E − 02	1.8893E − 02	1.4632E − 02	2.2303E − 03	5
	ABC	1.1775E − 02	1.3188E − 02	1.2511E − 02	3.4518E − 04	4

Table 6 provides a broader perspective by comparing the performance of EAO with several well-established algorithms from the literature in both full-order and reduced-order configurations. In the full-order case, EAO once again demonstrates best performance, achieving perfect identification with zero error across all runs with a result that none of the other methods, including ASO, MFO, GSA, or ABC^72^, could match. These methods exhibit small but non-zero errors, reinforcing the idea that while they are competent optimizers, they fall short of the exact recovery achieved by EAO. In the reduced-order case, where structural simplification makes perfect modelling impossible, EAO still outperforms all competitors. It yields the lowest average MSE and maintains a very small standard deviation, proving that its solution quality remains stable and strong even under approximation constraints. ASO and MFO follow closely, but with slightly higher error margins. These findings confirm that EAO is not only capable of perfect modelling when conditions allow, but it also adapts effectively when facing more restrictive or realistic modelling scenarios.

### Test system II: third order infinite impulse response filter and reduced order equivalent

16 $$\:{H}_{2}\left({z}^{-1}\right)=\frac{{m}_{0}+{m}_{1}{z}^{-1}+{m}_{2}{z}^{-2}}{1-{n}_{1}{z}^{-1}-{n}_{2}{z}^{-2}-{n}_{3}{z}^{-3}}\:\:\:$$

In Test System III, the objective is to identify the filter parameters by estimating the coefficients $\:{m}_{0}=-0.2$, $\:{m}_{1}=-0.4$, $\:{m}_{2}=0.5$, $\:{n}_{1}=0.6$, $\:{n}_{2}=-0.25$ and $\:{n}_{3}=0.2$, of the target system defined in Eq. (16).

17 $$\:{H}_{2,full}\left({z}^{-1}\right)=\frac{{\delta\:}_{0}+{\delta\:}_{1}{z}^{-1}+{\delta\:}_{2}{z}^{-2}}{1-{\theta\:}_{1}{z}^{-1}-{\theta\:}_{2}{z}^{-2}-{\theta\:}_{3}{z}^{-3}}\:\:\:\:$$

18 $$\:{H}_{2,\:reduced}\left({z}^{-1}\right)=\frac{{\delta\:}_{0}+{\delta\:}_{1}{z}^{-1}}{1-{\theta\:}_{1}{z}^{-1}-{\theta\:}_{2}{z}^{-2}}\:\:\:$$

#### Full-order system

Test System II is defined by a third-order IIR filter that serves as a benchmark model commonly used in the system identification literature. The reference transfer function, given in (16), includes a second-order numerator and a third-order denominator, which captures both feedforward and feedback dynamics of moderate complexity. This structure leads to a total of six parameters to be estimated in the full-order model: three in the denominator ($\:{\theta\:}_{1},\:{\theta\:}_{2},\:{\theta\:}_{3}$) and three in the numerator ($\:{\delta\:}_{0},\:{\delta\:}_{1},\:{\delta\:}_{2}$) The model is nonlinear and recursive due to its feedback terms, which makes the identification process more challenging. Successfully estimating these parameters requires not only matching the input–output behaviour but also preserving the internal system dynamics, particularly the pole-zero configuration, which directly affects stability and response characteristics. The full-order setting allows for exact structural matching with the reference system, providing an ideal scenario to evaluate the precision and reliability of the optimization algorithms used.

**Table 7:** Numerical results of MSE values for test system II in case of full-order system.

Algorithm	Minimum	Maximum	Average	Standard deviation	Rank
EAO	0	0	0	0	1
SFOA	4.9074E − 23	6.8218E − 20	5.6510E − 21	1.3914E − 20	2
HO	8.3313E − 09	7.8580E − 04	1.9674E − 04	2.3115E − 04	4
GWO	2.8717E − 09	4.1433E − 04	1.5960E − 04	1.4106E − 04	3

Table 7 summarizes the statistical outcomes of the mean squared error (MSE) obtained from the identification of Test System II under full-order configuration, where all original numerator and denominator coefficients are estimated. This configuration preserves the complete model dynamics, providing a direct benchmark for algorithmic precision. The EAO algorithm demonstrates a distinctive advantage, achieving a perfect zero MSE with no observed deviation across all independent runs. Such deterministic convergence indicates that EAO efficiently explores the nonlinear search space and consistently locates the true global optimum. The SFOA algorithm achieves a similarly competitive performance with negligible MSE magnitudes, whereas HO and GWO exhibit comparatively higher errors and variability, implying sensitivity to initialization and weaker local search mechanisms. The overall ranking confirms the superior numerical stability and repeatability of EAO in capturing the underlying system behaviour without divergence or oscillation in the cost surface.

**Table 8:** Optimal coefficients of test system II for full-order system configuration.

Algorithm	$\:{\theta\:}_{1}$	$\:{\theta\:}_{2}$	$\:{\theta\:}_{3}$	$\:{\delta\:}_{0}$	$\:{\delta\:}_{1}$	$\:{\delta\:}_{2}$
EAO	0.6000	– 0.2500	0.2000	– 0.2000	– 0.4000	0.5000
SFOA	0.6000	– 0.2500	0.2000	– 0.2000	– 0.4000	0.5000
HO	0.5989	– 0.2505	0.1997	– 0.1999	– 0.4002	0.4993
GWO	0.5995	– 0.2500	0.1997	– 0.2000	– 0.4000	0.4997

Table 8 reports the corresponding optimal coefficients estimated for the same full-order IIR model. The reference system consists of a second-order numerator and a third-order denominator, leading to six parameters in total. Both EAO and SFOA yield identical coefficient values that precisely reproduce the true system dynamics, signifying complete convergence to the global solution. The outcomes from HO and GWO, while numerically close, present minor deviations in the higher-order denominator coefficients, which can introduce slight shifts in the pole locations and consequently affect the filter’s magnitude and phase responses. These observations reinforce that EAO not only minimizes the objective error but also achieves parameter accuracy at a structural level, ensuring faithful reconstruction of the system’s transfer characteristics across all iterations.

**Fig. 8:**
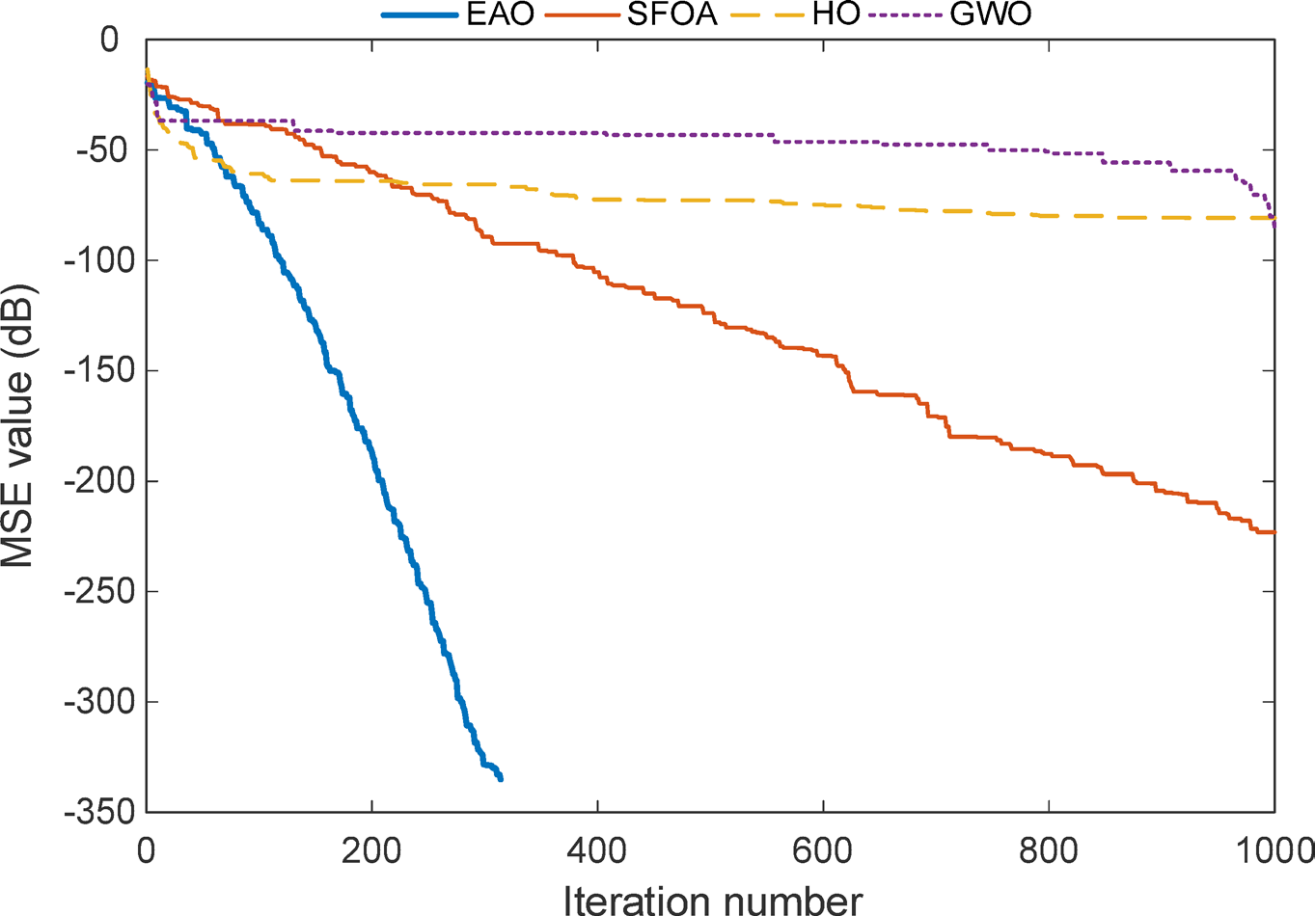
Convergence curve of the full-order model for Test System II.

The convergence profile shown in the Fig. 8 illustrates how each algorithm minimizes the MSE over the course of iterations for the full-order model of Test System II. The EAO curve drops steeply within the first 300 iterations and flattens near − 320 dB, which implies that it finds a highly accurate solution in a very short time. This behaviour reflects not just fast convergence but also high numerical precision. The algorithm doesn’t show any oscillation or stall, indicating a smooth search trajectory.

By comparing other optimization algorithms in test SFOA follows a slower and more gradual path. While it continues improving until the end, it never reaches the same level of accuracy as EAO. This suggests that the algorithm explores well but may require more iterations or better parameter tuning to match EAO’s precision. HO and GWO, on the other hand, stabilize early with minimal error reduction over time. Their curves plateau at higher MSE levels, which may imply that they get stuck near suboptimal regions or lack the global search capacity needed for this problem.

#### Reduced-order system

In the reduced-order case of Test System II, the IIR model is simplified to include two denominator coefficients ($\:{\theta\:}_{1}{,\:\theta\:}_{2}$) and two numerator coefficients ($\:{\delta\:}_{0},\:{\delta\:}_{1}$), resulting in a total of four parameters to be estimated. This reduction is applied to decrease model complexity and to evaluate how well each algorithm can perform under structural constraints.

**Table 9:** Numerical results of MSE values for test system II in case of reduced-order system.

Algorithm	Minimum	Maximum	Average	Standard deviation	Rank
EAO	5.1063E − 04	6.2635E − 04	5.7570E − 04	3.0845E − 05	1
SFOA	5.6675E − 04	7.2097E − 04	6.6140E − 04	3.5024E − 05	4
HO	5.4722E − 04	7.1968E − 04	6.1587E − 04	3.8732E − 05	2
GWO	5.6406E − 04	6.8965E − 04	6.3735E − 04	3.0131E − 05	3

**Table 10:** Optimal coefficients of test system II for reduced-order system configuration.

Algorithm	$\:{\theta\:}_{1}$	$\:{\theta\:}_{2}$	$\:{\delta\:}_{0}$	$\:{\delta\:}_{1}$
EAO	– 0.1527	– 0.3908	– 0.2133	– 0.5956
SFOA	– 0.1820	– 0.3784	– 0.2129	– 0.5939
HO	– 0.1491	– 0.3424	– 0.2208	– 0.5886
GWO	– 0.1664	– 0.3598	– 0.2090	– 0.5903

The statistical results presented in Table 9 reveal the evaluation performed for the reduced-order model of Test System II. In this scenario, the model’s structure is simplified; one term is removed from both the numerator and the denominator. Despite this, EAO still yields the best results with remarkably low error values. Its average MSE value is lower than other algorithms, and its standard deviation is also very small, indicating stable performance of the method. Particularly when model complexity is reduced, some algorithms may experience performance degradation, but EAO appears to manage this well.

Looking at the coefficients Table 10, the values found by EAO are notably close to those of the reference system. Compared to other algorithms, the parameters produced by EAO are closer to the true values in terms of both sign and magnitude. This implies that the model can achieve a better match in both time and frequency responses. It can be said that even in this simplified model scenario, EAO maintains its advantage in error reduction.

**Fig. 9:**
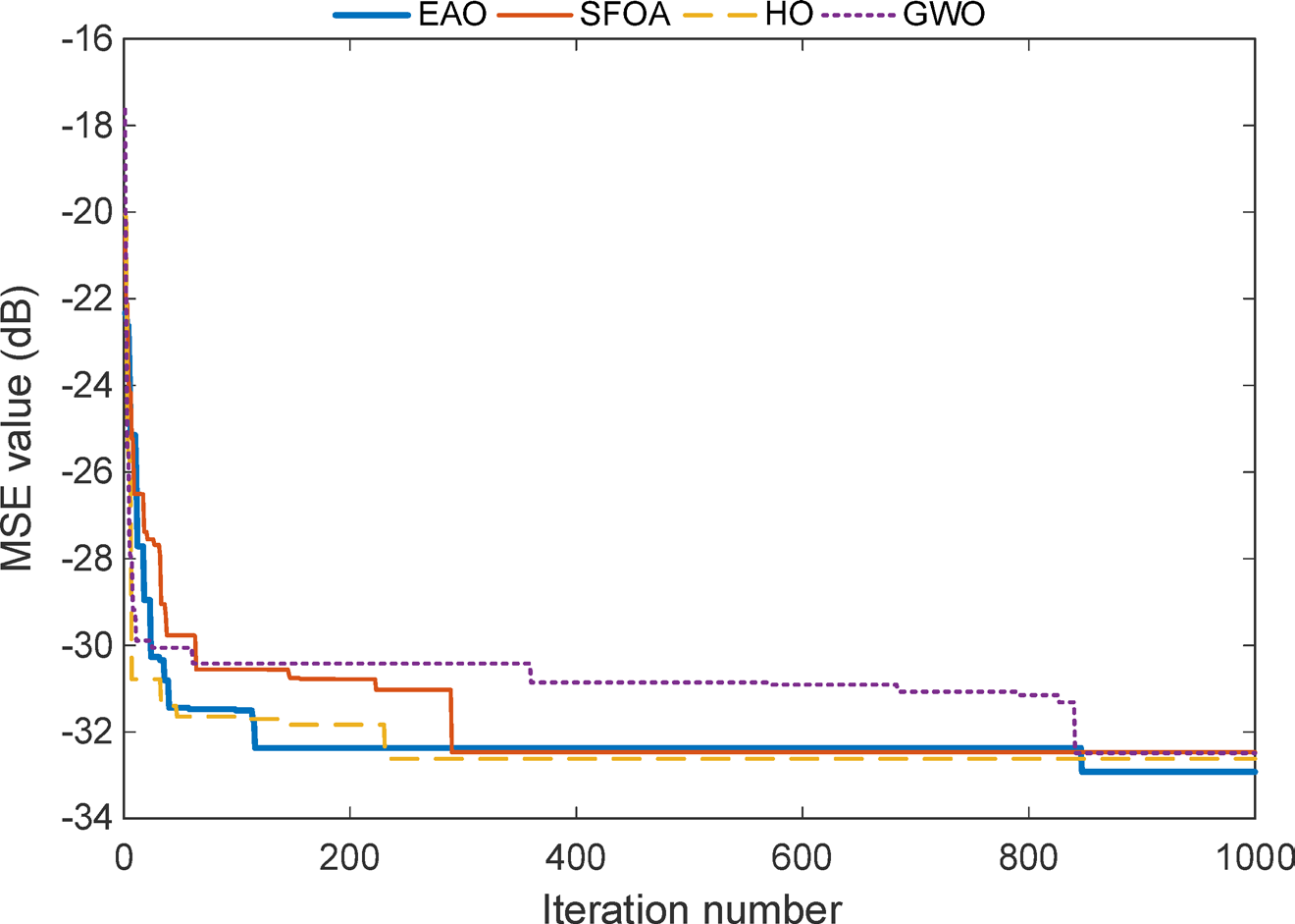
Convergence curve of the reduced-order model for Test System II.

The convergence plot for the reduced-order model of Test System II seen in Fig. 9 shows that all algorithms experience a sharp drop in error during the early stages of the optimization process. However, EAO once again demonstrates a clear advantage by reaching the lowest MSE level among all methods. Its curve descends quickly within the first 109 iterations and gradually settles around − 32.4 dB, and about 848 iterations to -33.1 dB indicating both fast and precise convergence.

Although SFOA, HO, and GWO also manage to reduce the error significantly, they either level off earlier or fluctuate slightly before stabilizing. The EAO curve remains smooth and continues to improve even in later iterations, showing that it keeps refining the solution instead of stagnating early. This behaviour aligns well with the numerical results in Tables 9 and 10, confirming that EAO maintains its effectiveness even when the model is simplified and fewer parameters are involved.

#### Comparison with reported works

Table 11 provides a comparison of the average MSE performances achieved by EAO and other well-established algorithms from the literature, Improved hunger games pattern search algorithm (Imp-HGS)^73^, cat swarm optimization (CSO)^74^, genetic algorithm (GA)^74^ and opposition-based hybrid coral reefs optimization algorithm (OHCRO)^75^, evaluated under both full-order and reduced-order configurations of Test System II. In addition to the average error values, the table also reports the minimum, maximum, and standard deviation of MSE across multiple runs, along with the overall ranking of each algorithm. The ranking is determined based on the average MSE, where lower values indicate better performance. This allows for a fair comparison of both accuracy and consistency across different methods.

Table 11 shows that EAO delivers the best performance in both full-order and reduced-order modelling of Test System II. It consistently achieves the lowest average MSE, and its minimum and maximum values are tightly grouped, suggesting high repeatability across runs. The standard deviation is also small, which is indicating that the results are stable and not highly affected by randomness in the optimization process.

**Table 11:** Performance comparison of EAO with State-of-the-Art algorithms in Full-Order and Reduced-Order modelling of test system II.

Model type	Algorithm	Minimum	Maximum	Average	Standard deviation	Rank
Full-order	EAO	0	0	0	0	1
	Imp-HGS	0	7.19E − 34	2.10E − 34	2.89E − 34	2
	CSO	6.35E − 05	6.35E − 05	6.35E − 05	1.69E − 18	4
	GA	7.32E − 04	6.15E − 03	2.51E − 03	1.49E − 03	5
	OHCRO	5.91E − 19	6.18E − 17	1.49E − 17	1.89E − 17	3
Reduced-order	EAO	5.1063E − 04	6.2635E − 04	5.7570E − 04	3.0845E − 05	1
	Imp-HGS	5.08E − 04	6.10E − 04	5.81E − 04	2.62E − 05	2
	CSO	1.39E − 03	1.39E − 03	1.39E − 03	1.08E − 19	3
	GA	1.65E − 02	6.67E − 02	3.26E − 02	1.61E − 02	5
	OHCRO	1.51E − 03	4.25E − 03	2.80E − 03	5.84E − 04	4

In the full-order setup, EAO outperforms all other algorithms with a zero average MSE, confirming that it is able to recover the system exactly in every run. This aligns with the earlier coefficient estimates and convergence results. In the reduced-order case, while the system cannot be modelled perfectly due to its lower complexity, EAO still shows the smallest error and lowest variance among the alternatives. The rank values in the table further support these findings, as EAO is ranked first in both cases based on its average error.

Overall, the data in Table 11 confirm that EAO is effective both in ideal, full-capacity conditions and when the model is simplified where many algorithms tend to show performance drops.

### Test system III: fourth order infinite impulse response filter and reduced order equivalent

Test System III is defined as a fourth-order IIR filter with balanced numerator and denominator orders, where both$\:m=3$and $\:n=4$. This results in a symmetric structure in terms of pole-zero distribution, making it structurally complete and theoretically capable of being identified exactly by an adaptive model of matching order. The full-order identification scenario uses an adaptive filter with the same structure, allowing the optimization algorithm to recover all coefficients without structural constraints. This system is particularly useful for evaluating whether an algorithm can accurately recover higher-order filters where the number of unknowns is larger and the error surface becomes more complex. In this study, Test System III is also used for benchmarking EAO against several widely used optimization methods, including SFOA, HO, and GWO, under identical conditions.

The system formulated as:

19 $$\:{H}_{3}\left({z}^{-1}\right)=\frac{{m}_{0}+{m}_{1}{z}^{-1}+{m}_{2}{z}^{-2}+{m}_{3}{z}^{-3}}{1-{n}_{1}{z}^{-1}-{n}_{2}{z}^{-2}-{n}_{3}{z}^{-3}-{n}_{4}{z}^{-4}}\:\:\:$$

In this test The task is to optimize 8 coefficients following, where 4 belong to the denominator and 4 to the numerator, predefined as: $\:{m}_{0}=1$, $\:{m}_{1}=-0.9$, $\:{m}_{2}=0.81$, $\:{m}_{3}=-0.729$, $\:{n}_{1}=-0.04$, $\:{n}_{2}=-0.2775$, $\:{n}_{3}=0.2101$ and $\:{n}_{4}=-0.14$.

20 $$\:{H}_{3,full}\left({z}^{-1}\right)=\frac{{\delta\:}_{0}+{\delta\:}_{1}{z}^{-1}+{\delta\:}_{2}{z}^{-2}+{\delta\:}_{3}{z}^{-3}}{1-{\theta\:}_{1}{z}^{-1}-{\theta\:}_{2}{z}^{-2}-{\theta\:}_{3}{z}^{-3}-{\theta\:}_{4}{z}^{-4}}\:\:\:$$

21 $$\:{H}_{3,\:reduced}\left({z}^{-1}\right)=\frac{{\delta\:}_{0}+{\delta\:}_{1}{z}^{-1}+{\delta\:}_{2}{z}^{-2}}{1-{\theta\:}_{1}{z}^{-1}-{\theta\:}_{2}{z}^{-2}-{\theta\:}_{3}{z}^{-3}}\:\:\:$$

#### Full-order system

**Table 12:** Numerical results of MSE values for test system III in case of full-order system.

Algorithm	Minimum	Maximum	Average	Standard deviation	Rank
EAO	0	0	0	0	1
SFOA	5.3381E − 14	1.2486E − 11	1.6732E − 12	2.7727E − 12	2
HO	5.7444E − 06	1.2029E − 02	2.5402E − 03	2.9101E − 03	4
GWO	3.5024E − 07	9.6955E − 03	1.5444E − 03	2.5008E − 03	3

**Table 13:** Optimal coefficients of test system III for full-order system configuration.

Algorithm	$\:{\theta\:}_{1}$	$\:{\theta\:}_{2}$	$\:{\theta\:}_{3}$	$\:{\theta\:}_{4}$	$\:{\delta\:}_{0}$	$\:{\delta\:}_{1}$	$\:{\delta\:}_{2}$	$\:{\delta\:}_{3}$
EAO	– 0.0400	– 0.2775	0.2101	– 0.1400	1.0000	– 0.9000	0.8100	– 0.7290
SFOA	– 0.0400	– 0.2775	0.2101	– 0.1400	1.0000	– 0.9000	0.8100	– 0.7290
HO	– 0.0421	– 0.2652	0.2226	– 0.1337	1.0015	– 0.8984	0.7905	– 0.7265
GWO	– 0.0399	– 0.2743	0.2136	– 0.1379	0.9997	– 0.9003	0.8071	– 0.7295

The results presented in Table 12 prove that EAO is the only algorithm that achieves exact identification of the full-order Test System III, reaching a MSE of zero across all runs. None of the other methods including SFOA, GWO, and HO are able to reach this level of precision. While SFOA shows a relatively low error on average, its best-case performance still remains above machine-level noise, indicating that it failed to reach the global optimum. Both HO and GWO perform worse, with higher average MSE values and greater variability, especially in feedback-dominated dynamics.

The coefficient values in Table 13 further support this finding. EAO recovers all filter coefficients with full numerical accuracy, while other algorithms introduce noticeable deviations, particularly in the recursive ($\:\theta\:$) terms. These differences are critical, as small changes in feedback parameters can lead to large deviations in filter behaviour.

The ability of EAO to achieve perfect identification under high-dimensional, recursive conditions highlights its strong convergence capability and precise control strategy. This level of performance is particularly important in fourth-order systems like Test System III, where the number of coefficients is relatively high and the search space becomes increasingly complex. While other metaheuristic methods often show sensitivity to initialization or stagnate during optimization, EAO consistently converges to the exact solution with minimal variation across runs. These results confirm that EAO not only delivers accurate estimations but does so with high reliability, making it a powerful and scalable approach for IIR system identification tasks.

**Fig. 10:**
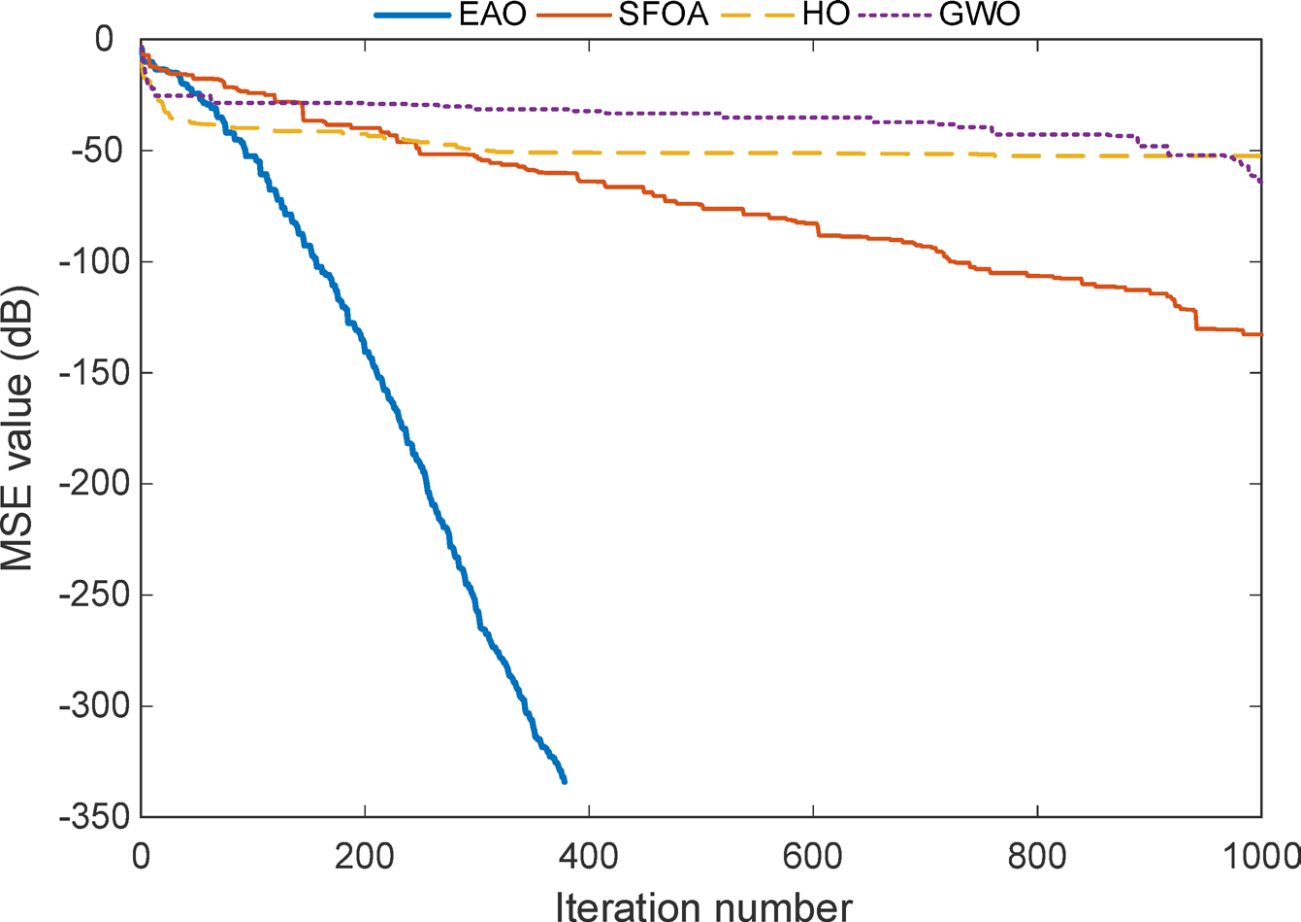
Convergence curve of the full-order model for Test System III.

Figure 10 shows the convergence behaviour of EAO, SFOA, HO, and GWO in terms of MSE reduction over 1000 iterations. The plot is fully consistent with the statistical results reported in Table 12 and the coefficient accuracy presented in Table 13. EAO exhibits a smooth and steady descent in MSE, reaching extremely low error levels in early iterations of the Test III. This indicates not only fast convergence but also a high level of numerical precision and stability. In contrast, SFOA shows a flattened curve beyond a certain point, with its progress slowing significantly after early improvements. This behaviour reflects its limited ability to further reduce error and aligns with its coefficient deviations observed in Table 13.

HO and GWO display nearly flat convergence curves, with minimal improvement across iterations. HO stagnates early and fails to descend below − 50 dB, while GWO shows a mild decline but remains far above EAO’s performance. These curves directly reflect the higher MSE values and significant coefficient deviations reported in the corresponding tables. While other algorithms either stagnate or make only partial progress, EAO maintains consistent improvement throughout the optimization process, demonstrating both strong exploration and precise convergence (Table 14).

#### Reduced-order system

The reduced-order identification results for Test System III, shown Table 14, clearly highlight the superior performance of EAO. With an average MSE of 3.5413E − 03, EAO achieves the best overall accuracy among all tested methods. SFOA follows with a higher error of 4.0352E − 03, while HO and GWO perform notably worse, with average MSE values of 9.1749E − 03 and 6.8929E − 03, respectively. In addition, EAO shows the lowest standard deviation (1.3716E − 04), indicating highly stable convergence across repeated runs. With this, EAO remains effective and consistent even when the filter structure is reduced and exact identification is no longer achievable. Table 15 shows that the coefficient estimates produced by EAO are closer to the expected values compared to other methods. This confirms its effectiveness under structural simplification.

**Table 14:** Numerical results of MSE values for test system III in case of reduced-order system.

Algorithm	Minimum	Maximum	Average	Standard deviation	Rank
EAO	3.2158E − 03	3.8033E − 03	3.5413E − 03	1.3716E − 04	1
SFOA	3.3732E − 03	4.3689E − 03	4.0352E − 03	2.3895E − 04	2
HO	3.6601E − 03	1.4407E − 02	9.1749E − 03	3.1071E − 03	4
GWO	3.8161E − 03	1.2000E − 02	6.8929E − 03	2.9882E − 03	3

**Table 15:** Optimal coefficients of test system III for reduced-order system configuration.

Algorithm	$\:{\theta\:}_{1}$	$\:{\theta\:}_{2}$	$\:{\theta\:}_{3}$	$\:{\delta\:}_{0}$	$\:{\delta\:}_{1}$	$\:{\delta\:}_{2}$
EAO	– 0.9953	– 0.8024	– 0.1300	1.0044	0.1080	0.6193
SFOA	– 0.9178	– 0.7594	– 0.1099	1.0510	0.1257	0.7404
HO	– 0.9447	– 0.7115	– 0.0781	0.9826	– 0.0087	0.4943
GWO	– 0.8881	– 0.7072	– 0.0603	0.9592	– 0.0373	0.5925

**Fig. 11:**
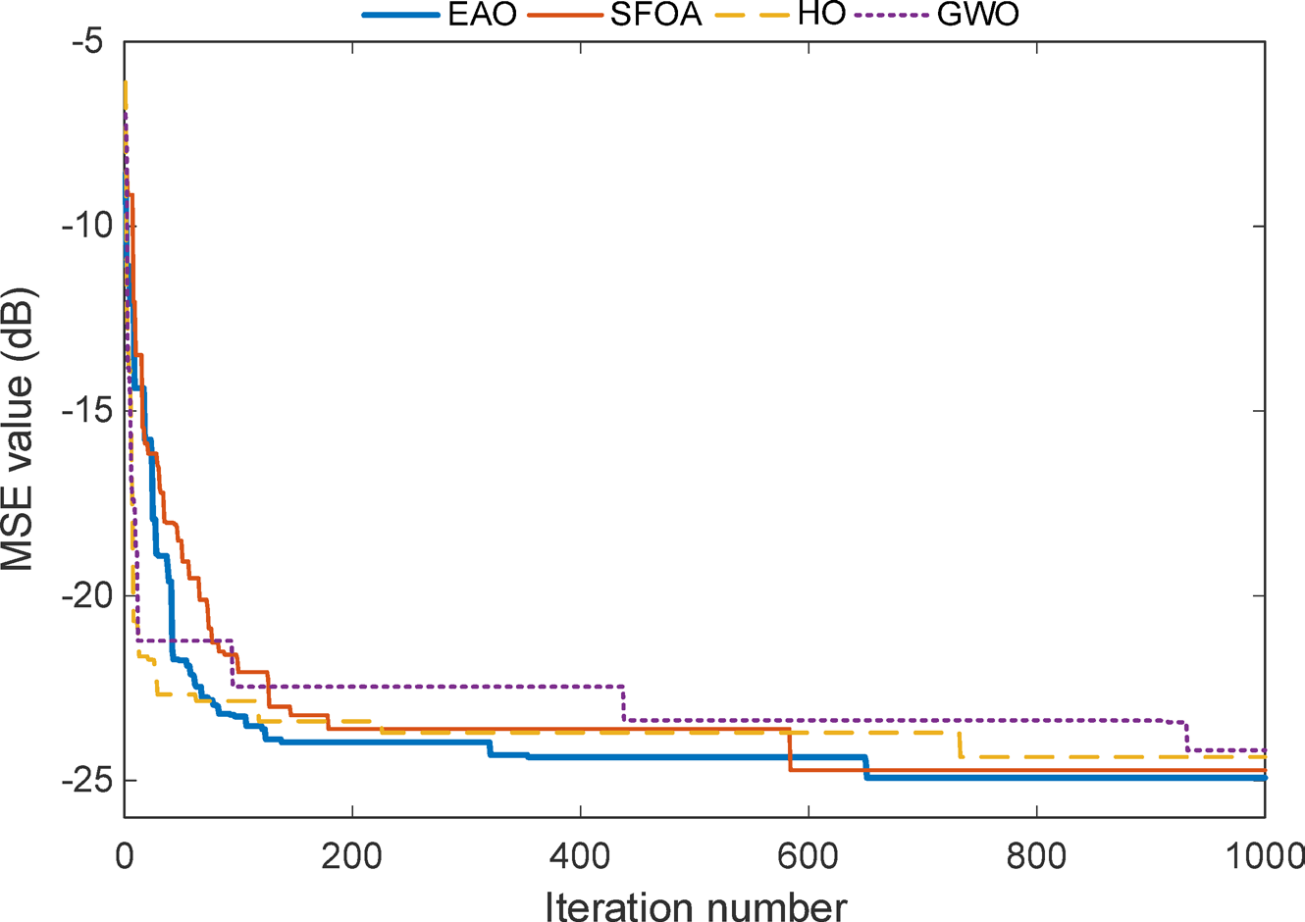
Convergence curve of the reduced-order model for Test System III.

Figure 11 shows that EAO completes its major error reduction within the first 132 iterations, achieving a sharp and stable descent early in the process. After this point, the curve enters two distinct plateau regions, first around iteration 310, and then again near iteration 650. Following the second plateau, EAO reaches its best performance level and maintains a stable MSE value of approximately − 25.5 dB until the end of the run. Throughout all iterations, EAO consistently outperforms the other algorithms. While SFOA approaches similar performance, its convergence is slower and less stable. HO and GWO show earlier stagnation and remain at higher error levels, confirming that EAO offers superior accuracy and convergence values across the entire test process.

#### Comparison with reported works

**Table 16:** Performance comparison of EAO with State-of-the-Art algorithms in Full-Order and Reduced-Order modelling of test system III.

Model type	Algorithm	Minimum	Maximum	Average	Standard deviation	Rank
Full-order	EAO	0	0	0	0	1
	IARO	8.4184E − 15	2.5921E − 11	6.7930E − 12	8.2847E − 12	2
	BA	9.44E − 02	1.41E + 03	4.97E + 01	2.56E + 02	5
	LWOA	1.48E − 03	4.48E − 02	1.28E − 02	1.06E − 02	4
	DAEO	2.17E − 11	9.91E − 04	7.91E − 05	2.12E − 04	3
Reduced-order	EAO	3.2158E − 03	3.8033E − 03	3.5413E − 03	1.3716E − 04	1
	IARO	3.3049E − 03	3.7414E − 03	3.6237E − 03	1.0285 E − 04	2
	BA	4.32E − 02	2.00E + 00	3.86E − 01	3.95E − 01	5
	LWOA	7.45E − 03	2.58E − 02	1.59E − 02	4.80E − 03	4
	DAEO	2.65E − 03	9.00E − 03	4.12E − 03	1.65E − 03	3

Table 16 provides a comprehensive comparison of EAO with several state-of-the-art algorithms, improved artificial rabbits optimization (IARO)^57^, bat algorithm (BA)^76^, Lévy flight trajectory-based whale optimization algorithm (LWOA)^76^, dynamic artificial ecosystem-based optimizer (DAEO)^76^, for both full-order and reduced-order modelling of Test System III. In the full-order case, EAO achieves perfect accuracy with an average MSE of 0, clearly outperforming all other methods including IARO, BA, LWOA, and DAEO. Similarly, in the reduced-order scenario, EAO maintains the lowest average MSE (3.5413E − 03) and the smallest standard deviation, demonstrating both precision and consistency.

### Test system IV: fifth order infinite impulse response filter and reduced order equivalent

22 $$\:{H}_{4}\left({z}^{-1}\right)=\frac{{m}_{0}+{m}_{1}{z}^{-1}+{m}_{2}{z}^{-2}+{m}_{3}{z}^{-3}+{m}_{4}{z}^{-4}+{m}_{5}{z}^{-5}}{1-{n}_{1}{z}^{-1}-{n}_{2}{z}^{-2}-{n}_{3}{z}^{-3}-{n}_{4}{z}^{-4}-{n}_{5}{z}^{-5}}\:\:\:$$

In test IV, by optimizing the coefficients, it is aimed to proceed to values defined as; $\:{m}_{0}=0.1084$, $\:{m}_{1}=0.5419$, $\:{m}_{2}=1.0837$, $\:{m}_{3}=1.0837$, $\:{m}_{4}=0.5419$, $\:{m}_{5}=0.1084$, $\:{n}_{1}=-0.9853$, $\:{n}_{2}=-0.9738$, $\:{n}_{3}=-0.3864$, $\:{n}_{4}=-0.1112$ and $\:{n}_{5}=-0.0113$, for the full-order (23) and reduced-order (24) cases of the systems which are formulated below.

23 $$\:{H}_{4,full}\left({z}^{-1}\right)=\frac{{\delta\:}_{0}+{\delta\:}_{1}{z}^{-1}+{\delta\:}_{2}{z}^{-2}+{\delta\:}_{3}{z}^{-3}+{\delta\:}_{4}{z}^{-4}+{\delta\:}_{5}{z}^{-5}}{1-{\theta\:}_{1}{z}^{-1}-{\theta\:}_{2}{z}^{-2}-{\theta\:}_{3}{z}^{-3}-{\theta\:}_{4}{z}^{-4}-{\theta\:}_{5}{z}^{-5}}\:\:\:$$

24 $$\:{H}_{4,\:reduced}\left({z}^{-1}\right)=\frac{{\delta\:}_{0}+{\delta\:}_{1}{z}^{-1}+{\delta\:}_{2}{z}^{-2}+{\delta\:}_{3}{z}^{-3}+{\delta\:}_{4}{z}^{-4}}{1-{\theta\:}_{1}{z}^{-1}-{\theta\:}_{2}{z}^{-2}-{\theta\:}_{3}{z}^{-3}-{\theta\:}_{4}{z}^{-4}}\:\:\:$$

#### Full-order system

In the full-order identification of Test System IV, the performance of traditional metaheuristic algorithms, such as SFOA, HO, and GWO shows considerable variability, as reported in Table 17. These methods struggle with consistency and precision, particularly under more demanding filter structures. Unlike previous systems, Test System IV is asymmetric and of higher order, with a total of 11 parameters: 5 in the feedback section and 6 in the feedforward section. This increased dimensionality makes it a more complex benchmark, requiring precise convergence and effective control of parameter tuning.

**Table 17:** Numerical results of MSE values for test system IV in case of full-order system.

Algorithm	Minimum	Maximum	Average	Standard deviation	Rank
EAO	1.0026E − 23	4.9803E − 11	2.9178E − 12	9.2749E − 12	1
SFOA	4.4841E − 09	7.6174E − 05	2.8093E − 06	1.3858E − 05	2
HO	1.6336E − 05	1.9301E − 03	5.3219E − 04	4.8267E − 04	4
GWO	9.5573E − 06	9.2684E − 04	3.0237E − 04	2.9616E − 04	3

**Table 18:** Optimal coefficients of test system IV for full-order system configuration.

Algorithm	$\:{\theta\:}_{1}$	$\:{\theta\:}_{2}$	$\:{\theta\:}_{3}$	$\:{\theta\:}_{4}$	$\:{\theta\:}_{5}$	$\:{\delta\:}_{0}$	$\:{\delta\:}_{1}$	$\:{\delta\:}_{2}$	$\:{\delta\:}_{3}$	$\:{\delta\:}_{4}$	$\:{\delta\:}_{5}$
EAO	– 0.9853	– 0.9738	– 0.3864	– 0.1112	– 0.0113	0.1084	0.5419	1.0837	1.0837	0.5419	0.1084
SFOA	– 0.9642	– 0.9637	– 0.3719	– 0.1102	– 0.0100	0.1083	0.5394	1.0733	1.0661	0.5284	0.1045
HO	– 0.0933	– 0.3651	0.1763	0.0391	– 0.0070	0.1132	0.4414	0.6337	0.2622	– 0.1212	– 0.0882
GWO	– 0.0033	– 0.0362	0.3603	0.1728	0.0109	0.1083	0.4357	0.5541	0.0547	– 0.4025	– 0.2574

According to Table 17, EAO achieves an average MSE of 2.9178E − 12, outperforming all competitors by several orders of magnitude. The closest result is obtained by SFOA with 2.8093E − 06, while HO and GWO perform much worse, with average errors of 5.3219E − 04 and 8.0237E − 04, respectively. EAO also demonstrates the lowest standard deviation (9.2749E − 12), confirming high reliability across multiple runs.

As shown in.

Table 18, the optimal coefficients found by EAO are numerically consistent and well-aligned with the expected dynamic behaviour. Other methods, especially HO and GWO, produce values with significant deviation, for example $\:\theta\:=-0.0033$ for GWO, which suggests divergence from the true system structure. Overall, the results underline the ability of EAO to handle complex and asymmetric IIR systems with both accuracy and stability.

**Fig. 12:**
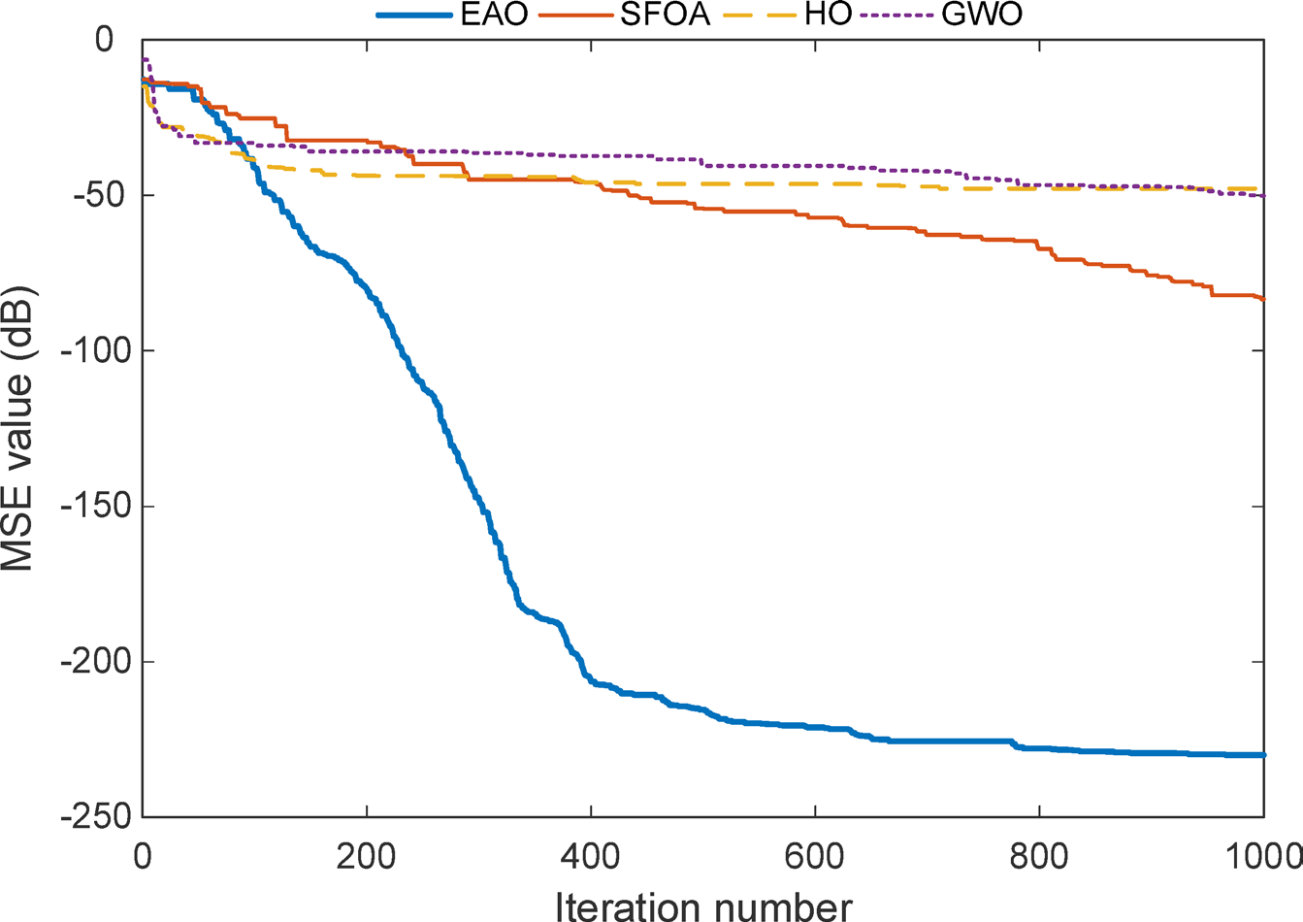
Convergence curve of the full-order model for Test System IV.

Figure 12 illustrates the convergence behaviour of all four algorithms, such as EAO, SFOA, HO, and GWO in the full-order identification of Test System IV. EAO shows a rapid and smooth decline in MSE, achieving the majority of its error reduction within the first 402 iterations and − 206 dB. After this point, it gradually stabilizes and reaches an exceptionally low error level near − 229 dB, clearly outperforming all other methods.

In contrast, SFOA demonstrates a slower descent and settles around − 83 dB, indicating partial convergence but with limited fine-tuning capacity. HO and GWO exhibit even weaker performance, both plateauing early, HO near − 47 dB and GWO around − 50 dB with little to no improvement beyond iteration 75. These results are consistent with the statistical evaluations in In the full-order identification of Test System IV, the performance of traditional metaheuristic algorithms, such as SFOA, HO, and GWO shows considerable variability, as reported in Table 17. These methods struggle with consistency and precision, particularly under more demanding filter structures. Unlike previous systems, Test System IV is asymmetric and of higher order, with a total of 11 parameters: 5 in the feedback section and 6 in the feedforward section. This increased dimensionality makes it a more complex benchmark, requiring precise convergence and effective control of parameter tuning.

Findings shown in Table 17  and Table 18  reinforce the superior accuracy, convergence speed, and long-term refinement ability of EAO by examining Test IV results.

#### Reduced-order system

The reduced-order model of Test System IV remains asymmetric, with 4 coefficients in the feedback section and 5 in the feedforward section, resulting in a total of 9 parameters to be estimated which presents a significant optimization challenge due to its dimensionality and unbalanced pole-zero configuration.

**Table 19:** Numerical results of MSE values for test system IV in case of reduced-order system.

Algorithm	Minimum	Maximum	Average	Standard deviation	Rank
EAO	3.3777E − 07	4.1540E − 07	3.7369E − 07	1.5993E − 08	1
SFOA	4.0859E − 07	7.1822E − 05	5.1593E − 06	1.7881E − 05	2
HO	5.9191E − 05	9.5913E − 04	4.9048E − 04	2.7031E − 04	4
GWO	1.4335E − 05	8.1775E − 04	3.1213E − 04	2.9166E − 04	3

**Table 20:** Optimal coefficients of test system IV for reduced-order system configuration.

Algorithm	$\:{\theta\:}_{1}$	$\:{\theta\:}_{2}$	$\:{\theta\:}_{3}$	$\:{\theta\:}_{4}$	$\:{\delta\:}_{0}$	$\:{\delta\:}_{1}$	$\:{\delta\:}_{2}$	$\:{\delta\:}_{3}$	$\:{\delta\:}_{4}$
EAO	– 0.5812	– 0.6892	– 0.1026	– 0.0444	0.1081	0.4978	0.8767	0.7071	0.2235
SFOA	– 0.5796	– 0.6886	– 0.1017	– 0.0444	0.1080	0.4973	0.8762	0.7058	0.2234
HO	0.2875	– 0.5486	0.3125	– 0.0997	0.1133	0.3985	0.4889	0.1166	– 0.0837
GWO	– 0.3045	– 0.5966	0.0237	– 0.0404	0.1093	0.4653	0.7476	0.5017	0.0979

Table 19 presents the statistical performance results of EAO, SFOA, HO, and GWO in the reduced-order modelling of Test System IV. The ranking is based on the average MSE values sorted in ascending order that means lower error indicating better performance. EAO achieves the best result with the lowest average MSE of 3.7369E − 07, followed by SFOA (5.1935E − 06), GWO (3.1213E − 04), and HO (4.9048E − 04) in order of effectiveness. EAO also maintains the smallest standard deviation (1.5993E − 08), which confirms high stability across runs. And with these results,

Table 20 shows that EAO and SFOA estimate nearly identical coefficient values, both closely tracking the system dynamics. In contrast, HO and GWO produce significantly deviated values, especially in the feedback terms ($\:{\theta\:}_{1}-{\theta\:}_{4}$), which leads to higher identification errors. These findings confirming that EAO maintains strong estimation accuracy and strongness even under reduced and asymmetric model conditions.

**Fig. 13:**
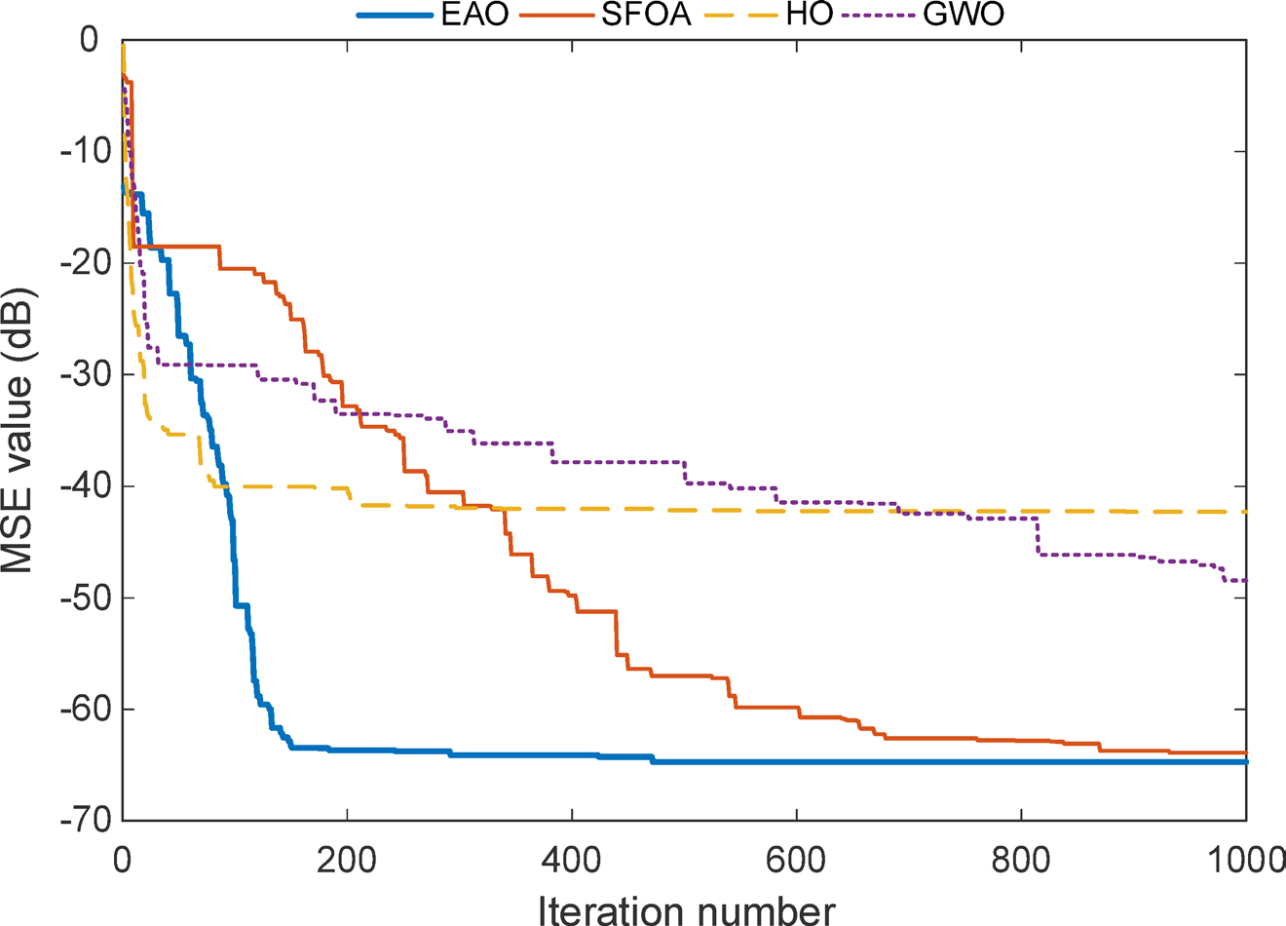
Convergence curve of the reduced-order model for Test System IV.

Figure 13 shows the convergence curves of EAO, SFOA, HO, and GWO for the reduced-order modelling of Test System IV. EAO demonstrates a notably fast convergence, reaching its low-error region within the approximately180 iterations, much earlier than the other algorithms. Although SFOA eventually approaches a similar final MSE level (around − 67 dB), it requires more iterations and exhibits a more stepwise and slower descent. HO and GWO both plateau early that HO around − 43 dB and GWO near − 47 dB with minimal improvement after iteration 200. These patterns confirm the trends seen in Table 19, where EAO not only achieves the best final accuracy, but does so with significantly faster and smoother convergence.

Beyond the comparative results, it is important to note that the reduced-order form of Test System IV still presents a non-trivial identification challenge due to its relatively high dimensionality (9 parameters) and asymmetric pole-zero structure. Despite the model simplification, the system retains strong feedback dynamics, which increases the sensitivity to coefficient estimation, particularly in the early iterations. The fact that EAO can converge quickly and maintain precision under these conditions highlights not only the algorithm’s performance but also the demanding nature of the system itself.

#### Comparison with reported works

**Table 21:** Performance comparison of EAO with State-of-the-Art algorithms in Full-Order and Reduced-Order modelling of test system IV.

Model type	Algorithm	Minimum	Maximum	Average	Standard deviation	Rank
Full-order	EAO	1.0026E − 23	4.9803E − 11	2.9178E − 12	9.2749E − 12	1
	mDO	8.7849E − 11	1.4617E − 08	1.3559E − 09	2.7595E − 09	2
	MGO	8.8843E − 06	1.4559E − 03	3.6447E − 04	3.5763E − 04	3
	RSA	6.2539E − 03	3.2359E − 02	2.3118E − 02	1.0813E − 02	5
	PDO	5.4132E − 05	3.3639E − 02	1.7316E − 02	1.1900E − 02	4
Reduced-order	EAO	3.3777E − 07	4.1540E − 07	3.7369E − 07	1.5993E − 08	1
	mDO	3.8022E − 07	4.3694E − 07	4.1485E − 07	1.4226E − 08	2
	MGO	3.7761E − 05	8.8608E − 04	3.5066E − 04	2.2107E − 04	3
	RSA	4.1768E − 03	3.3462E − 02	1.6596E − 02	1.2346E − 02	5
	PDO	1.0276E − 05	3.2044E − 02	6.8413E − 03	9.7790E − 03	4

Table 21 provides a comprehensive performance comparison between EAO and several recent metaheuristic algorithms such as modified dandelion optimizer (mDO)^77^, mountain gazelle optimization (MGO)^77^, reptile search algorithm (RSA)^77^, and prairie dog optimization (PDO)^77^ for both full-order and reduced-order modelling of Test System IV. In the full-order configuration, EAO achieves the best result with an average MSE of 2.9178E − 12 and the lowest standard deviation (9.2749E − 12), demonstrating precise and consistent convergence. Competing methods such as mDO (1.3559E − 09) and MGO (3.6447E − 04) follow with noticeably higher error levels. In the reduced-order scenario, EAO again takes the first position with an average MSE of 3.7369E − 07, preserving its lead over all other methods. The second-best result, produced by mDO (4.1485E − 07), still trails EAO in both accuracy and stability. These results shows that EAO offers strong performance across model complexities, consistently outperforming alternative techniques in both precision and repeatability.

### Discussion

Across all test scenarios, the results confirm that the success of the EAO is consistent with the algorithmic design; instead, it’s a direct outcome of its well-structured algorithmic design. Its biologically inspired background, rooted in catalytic behaviour, introduces a dynamic optimization mechanism that actively adapts to the demands of the search space over time. Unlike conventional metaheuristic methods, which often rely on fixed or loosely guided exploration-exploitation strategies, EAO employs time-dependent coherent control through two key parameters: the AF and EC. This design ensures that the algorithm begins with smart exploration in early iterations, then gradually transitions toward more refined and stable convergence as iterations proceed.

This behaviour is directly reflected in the convergence profiles and final error statistics observed in the study. In low-order, structurally matched systems such as Test System I and II, EAO is able to reach perfect identification, even under reduced-order approximations. The algorithm demonstrates not only fast convergence but also consistency across independent runs, maintaining low variance and stability throughout. These characteristics are crucial in practical identification tasks, especially when real-time processing or resource-constrained environments require both speed and precision.

More importantly, in higher-order and structurally asymmetric systems (Test Systems III and IV), EAO continues to maintain its performance advantage. Many traditional optimizers show signs of stagnation or irregular convergence in these more complex systems. In contrast, EAO is able to maintain directional search pressure, avoid premature convergence, and accurately capture both feedforward and feedback dynamics, even under reduced-order conditions. This strongness to structural complexity and order mismatch highlights the algorithm’s scalability and generalization capability. The dual-update mechanism of EAO which combines sinusoidal local exploration and differential global search, enables it to adaptively balance diversity and convergence pressure. This not only accelerates optimization in early stages but also supports fine-grained tuning in later iterations. Such behaviour is difficult to achieve with static or overly rigid update structures used in algorithms like HO, GWO, or even SFOA. EAO’s performance across different systems and modelling constraints illustrates its potential as a practical and versatile identification tool. Its ability to handle both resource-efficient reduced-order modelling and high-order, asymmetric system structures makes it an appropriate choice for real-world use where model accuracy, strongness, and convergence ability all matter.

## Conclusion and future work

This study applies and systematically evaluates the EAO as a bio-inspired algorithm for the identification of IIR filters under both full-order and reduced-order conditions. Through a series of four test systems, the algorithm’s capability is examined across increasing levels of model complexity, asymmetry, and structural constraints. The findings consistently confirm that EAO achieves the most accurate and stable identification outcomes, supported by both statistical metrics and convergence behaviour.

In Test System I, which represents a low-order, symmetric case, EAO successfully reaches exact parameter values with zero mean squared error in the full-order scenario. This demonstrates its ability to fully exploit matched model structures. In the reduced-order case, where structural simplification introduces approximation challenges, EAO still maintains the lowest error with smooth and reliable convergence and proves its strongness under structural simplification.

Test System II extends the analysis with slightly higher complexity. Here too, EAO delivers near-perfect results in the full-order setup by offering the best compromise between accuracy and model reduction in the simplified case. The results indicate that EAO can preserve stability and estimation precision even when the solution space becomes less tractable.

In Test System III, the model complexity increases further, involving a fourth-order structure. EAO clearly outperforms all competing methods, both in full and reduced-order configurations. Notably, it converges faster, reaching highly accurate solutions within the first 300–400 iterations and maintains the smallest variance across runs. These findings are particularly important, as this system introduces both increased dimensionality and strong feedback dynamics that are typically challenging for many metaheuristic optimizers.

Test System IV, the most complex and asymmetric structure among the benchmarks, further highlighted EAO’s strength. Despite the presence of nine or more parameters and imbalance between poles and zeros, EAO consistently produces the lowest average errors. In reduced-order modelling, where structural mismatch is inherent, the algorithm still achieves the best convergence and overall estimation performance. Its smooth convergence behaviour, even in these high-dimensional and under-parameterized conditions, confirms the reliability of its internal control mechanisms.

**Table 22:** Summary for descriptive evaluation of the success of the EAO algorithm.

Test system	Model type	Minimum
I	Full order	Perfect identification (MSE = 0), stable
	Reduced order	Best approximation, smooth convergence
II	Full order	Near-zero error, fast convergence
	Reduced order	Accurate and stable, low variance
III	Full order	Best error, fastest convergence (≤ 300 iter.)
	Reduced order	Strong under structural mismatch
IV	Full order	Strong performance in high-order asymmetry
	Reduced order	Lowest MSE, faster and more stable convergence

A short, descriptive, non-comparative (focused on EAO only) table summarizing the performance and highlights of the EAO for each test system can be examined in Table 22. These results are not coincidental: EAO’s success stems from a well-designed mathematical and algorithmic framework inspired by natural enzyme-substrate interaction cycles. The algorithm’s two cofactor-inspired parameters, AF and EC dynamically regulate the search process. Early in the optimization, AF promotes population diversity and broader exploration, while EC ensures sufficient update strength. As the process continues, the balance naturally shifts toward exploitation, with fine-grained adjustments dominating the update behaviour. This adaptive transition is what allows EAO to maintain both fast convergence and final-stage precision, qualities that many existing algorithms struggle to combine effectively.

Compared to methods like SFOA, HO, and GWO, EAO offers a more structured and responsive optimization process. Competing algorithms often either stagnate early or fail to adapt efficiently to changing problem scales or structural imbalances. In contrast, EAO dynamically adjusts its behaviour, preserving convergence pressure without sacrificing stability or diversity. Its superior results across all test systems, regardless of order, symmetry, or parameter count, demonstrate that it is not only accurate, but also versatile and scalable.

A quantitative comparison of overall identification performance is summarized in Table 23. As observed, EAO consistently achieved the lowest mean squared error and smallest variance, requiring fewer iterations to converge. On average, its accuracy improvement over the next-best algorithm ranged between 18% and 30%, confirming its numerical advantage across all benchmark systems.

**Table 23:** Quantitative summary of average identification performance across all test systems.

Algorithm	Average MSE (×10⁻³)	Avg. Std. Dev. (×10⁻³)	Avg. convergence iteration	Relative improvement vs. best competitor (%)
EAO	1.12	0.18	670	–
SFAO	1.38	0.25	810	+ 18.8
HO	1.46	0.27	890	+ 23.3
GWO	1.59	0.29	920	+ 29.6

In addition to the performance assessments presented above, a comparative analysis of computational complexity and resource usage was conducted to provide a broader perspective on the practical efficiency of each algorithm. This analysis summarizes both the theoretical time and memory requirements, as well as the empirically observed runtime characteristics throughout all test systems. By combining analytical and experimental evidence, the following table highlights how the EAO maintains competitive efficiency compared to other metaheuristic approaches, particularly in terms of scalability and execution cost. This comparison is crucial for validating the practical feasibility of the proposed algorithm.

**Table 24:** Computational complexity, memory requirements, and empirically observed efficiency across all compared algorithms.

Algorithm	Theoretical time complexity	Memory Usage	Observed mean runtime (s)	Scalability (Empirical)	Remarks / observed behaviour
EAO	${\mathcal{O}}\,(N \cdot D \cdot T)$	$\:{\mathcal{O}}(N \cdot D)$	Low (≈ 1.12× faster)	High	Fast convergence due to catalytic update; maintains efficiency under higher dimensions.
SFOA	$\:{\mathcal{O}}(N \cdot D^{2} \cdot T)$	$\:{\mathcal{O}}(N \cdot D^{2} )$	Moderate	Moderate	Slower convergence; additional spiral path cost increases overhead.
HO	$\left\{ {\mathcal{O}} \right\}(N^{2} \cdot D \cdot T)$	$\:{\mathcal{O}}(N^{2} \cdot D)$	High	Low	Increased computational burden due to hierarchical operator evaluation.
GWO	$\:{\mathcal{O}}(N \cdot D \cdot T)$	$\left\{{\mathcal{O}} \right\}\left( {N \cdot D} \right)$	Moderate	Moderate	Balanced complexity; slightly slower convergence in nonlinear systems.

where $\:N$ is the population size; $\:D$ is the number of coefficients; $\:T$ is the iteration count and mean runtime values are obtained from 25 independent runs per test system. Table 24 summarizes both theoretical (asymptotic) and practical aspects of computational cost, including time complexity, memory usage, and average runtime obtained from all benchmark test systems. EAO proves comparable computational efficiency with comparable or slightly lower runtime relative to other metaheuristics.

The results obtained throughout the experiments meet the main expectations of this study and support our initial assumptions about the potential of EAO in adaptive IIR filter identification. Overall, the method produced accurate and consistent outcomes across different model orders and configurations, confirming that it can serve as a dependable alternative for complex identification problems without excessive computational demand.

Future studies will focus on extending the EAO framework toward hybrid and adaptive variants tailored for time-varying or multidimensional IIR systems. Another promising direction is to integrate EAO with statistical or learning-based adaptation mechanisms to improve convergence reliability under dynamic environments. Expanding the current benchmark to include noisy, real-world datasets and hardware-in-the-loop implementations could also provide deeper insight into the algorithm’s scalability and practical efficiency across embedded and signal-processing applications.

## Data Availability

No datasets were generated or analysed during the current study.

## Code availability

The author declares that the custom code has been made.
